# The LIDPAD Mouse Model Captures the Multisystem Interactions and Extrahepatic Complications in MASLD

**DOI:** 10.1002/advs.202404326

**Published:** 2024-07-01

**Authors:** Zun Siong Low, Damien Chua, Hong Sheng Cheng, Rachel Tee, Wei Ren Tan, Christopher Ball, Norliza Binte Esmail Sahib, Ser Sue Ng, Jing Qu, Yingzi Liu, Haiyu Hong, Chaonong Cai, Nandini Chilagondanahalli Lakshmi Rao, Aileen Wee, Mark Dhinesh Muthiah, Zoë Bichler, Barbara Mickelson, Mei Suen Kong, Vanessa Shiyun Tay, Zhuang Yan, Jiapeng Chen, Aik Seng Ng, Yun Sheng Yip, Marcus Ivan Gerard Vos, Nicole Ashley Tan, Dao Liang Lim, Debbie Xiu En Lim, Manesh Chittezhath, Jadegoud Yaligar, Sanjay Kumar Verma, Harish Poptani, Xue Li Guan, Sambasivam Sendhil Velan, Yusuf Ali, Liang Li, Nguan Soon Tan, Walter Wahli

**Affiliations:** ^1^ Lee Kong Chian School of Medicine Nanyang Technological University Singapore Clinical Sciences Building, 11 Mandalay Road Singapore 308232 Singapore; ^2^ Metabolic Imaging Group Institute of Bioengineering and Bioimaging Agency for Science Technology and Research (A*STAR) 11 Biopolis Way Singapore 138667 Singapore; ^3^ Department of Pathogen Biology Shenzhen Center for Disease Control and Prevention Shenzhen 518055 China; ^4^ Intervention and Cell Therapy Center Peking University Shenzhen Hospital Shenzhen 518036 China; ^5^ Department of Otolaryngology Head and Neck Surgery The Fifth Affiliated Hospital of Sun Yat‐sen University 52 Mei Hua East Road Zhuhai 519000 China; ^6^ Department of Pathology Tan Tock Seng Hospital 11 Jalan Tan Tock Seng Singapore 308433 Singapore; ^7^ Department of Pathology National University Hospital 5 Lower Kent Ridge Rd Singapore 119074 Singapore; ^8^ Department of Medicine Yong Loo Lin School of Medicine National University of Singapore Singapore 117597 Singapore; ^9^ Division of Gastroenterology and Hepatology Department of Medicine National University Hospital Singapore 119074 Singapore; ^10^ National University Centre for Organ Transplantation National University Health System Singapore 119074 Singapore; ^11^ ENVIGO Madison WI 53713 USA; ^12^ Radcliffe Department of Medicine John Radcliffe Hospital University of Oxford Oxford OX3 9DU UK; ^13^ School of Biological Sciences Nanyang Technological University Singapore 60 Nanyang Drive Singapore 637551 Singapore; ^14^ Singapore Institute for Clinical Sciences A*STAR 30 Medical Drive Singapore 117609 Singapore; ^15^ Centre for Preclinical Imaging Institute of Systems Molecular & Integrative Biology University of Liverpool Biosciences Building, Crown Street Liverpool L69 7BE UK; ^16^ Singapore Eye Research Institute (SERI) Singapore General Hospital Singapore 168751 Singapore; ^17^ Department of Pharmacology School of Medicine Southern University of Science and Technology Shenzhen 518055 China; ^18^ Institut national de recherche pour l'agriculture l'alimentation et l'environnement (INRAE) Toxalim (Research Centre in Food Toxicology) 180 Chemin de Tournefeuille Toulouse 1331 France; ^19^ Center for Integrative Genomics Université de Lausanne Le Génopode Lausanne 1015 Switzerland; ^20^ Present address: The Jackson Laboratory 600 Main Street Bar Harbor ME 04609 USA

**Keywords:** diet‐induced weight loss, gut microbiome, human MASLD transcriptomic signature, MASH, MASLD

## Abstract

Metabolic dysfunction‐associated steatotic liver disease (MASLD) represents an impending global health challenge. Current management strategies often face setbacks, emphasizing the need for preclinical models that faithfully mimic the human disease and its comorbidities. The liver disease progression aggravation diet (LIDPAD), a diet‐induced murine model, extensively characterized under thermoneutral conditions and refined diets is introduced to ensure reproducibility and minimize species differences. LIDPAD recapitulates key phenotypic, genetic, and metabolic hallmarks of human MASLD, including multiorgan communications, and disease progression within 4 to 16 weeks. These findings reveal gut‐liver dysregulation as an early event and compensatory pancreatic islet hyperplasia, underscoring the gut‐pancreas axis in MASLD pathogenesis. A robust computational pipeline is also detailed for transcriptomic‐guided disease staging, validated against multiple harmonized human hepatic transcriptomic datasets, thereby enabling comparative studies between human and mouse models. This approach underscores the remarkable similarity of the LIDPAD model to human MASLD. The LIDPAD model fidelity to human MASLD is further confirmed by its responsiveness to dietary interventions, with improvements in metabolic profiles, liver histopathology, hepatic transcriptomes, and gut microbial diversity. These results, alongside the closely aligned changing disease‐associated molecular signatures between the human MASLD and LIDPAD model, affirm the model's relevance and potential for driving therapeutic development.

## Graphical abstract

The liver disease progression aggravation diet is a single mouse model with a refined diet that rapidly replicates the full metabolic dysfunction‐associated steatotic liver disease (MASLD) spectrum in 16 weeks under thermoneutral conditions. It captures key pathogenic features like metabolic disorders, gut dysbiosis, multi‐organ dysfunction, neurobehavioral anomalies, and hepatic transcriptomic shifts, making it highly relevant for advancing MASLD translational research.

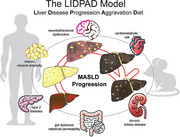

## 1 Introduction

With metabolic dysfunction‐associated steatotic liver disease (MASLD)^[^ ^1^ ^]^ impacting an estimated 30% of the global population,^[^ ^2^ ^]^ and metabolic dysfunction‐associated steatohepatitis (MASH) occurring in ≈10–30% of these patients,^[^ ^3^ ^]^ the situation calls for urgent attention. The prevalence of these conditions is projected to escalate in line with the intensifying epidemic of metabolic syndrome components such as obesity and diabetes mellitus.^[^ ^4^ ^]^ Without appropriate intervention, MASH patients are likely to advance to end‐stage liver disease and face heightened cardiovascular disease risk.^[^ ^5^ ^]^ Existing management primarily focuses on weight loss, but this approach is often met with setbacks as ≈80% of patients experience weight regain within one year, exacerbating fibrotic severity and escalating mortality rates in recurrent MASH cases.^[^ ^6^ ^]^ By the time the disease advances to cirrhosis, these measures lose their effectiveness.^[^ ^7^ ^]^ Resmetirom (Rezdiffra), the first FDA‐approved oral thyroid hormone receptor‐*β* agonist for MASH, represents an important yet measured advance. In its phase III trial, resmetirom's placebo‐subtracted effect showed a 16.4–20.7% improvement in MASH resolution and a 10.2–11.8% improvement in fibrosis.^[^ ^8^ ^]^ This translates to ≈2 in 10 patients achieving MASH resolution and 1 in 10 experiencing fibrosis improvement with treatment. Despite these promising results, challenges remain, including its limited efficacy for the majority of patients and potential side effects,^[^ ^9^ ^]^ underscoring the importance of long‐term patient monitoring, and the pursuit of other pharmacological options due to the complex nature of the disease. Indeed, human MASLD is more than just a liver disease. Majority of MASLD patients often present with other serious health issues such as cardiovascular disease, type‐II diabetes, and chronic kidney disease. However, our understanding of the inter‐organ mechanisms driving these diseases is distressingly inadequate, underlining the urgency need for more research and improved treatment strategies.

To enhance our understanding of MASH and to facilitate the development of effective treatments, it is crucial to use preclinical models that accurately reflect human disease features and comorbidities.^[^ ^10^ ^]^ Thermoneutral housing has emerged as a critical element in preclinical rodent models due to its ability to closely mirror human metabolic conditions by eliminating cold‐induced thermogenesis and/or non‐shivering thermogenesis.^[^ ^11^ ^]^ However, initial studies using thermoneutral housing and a high‐fat diet (HFD) in mice indicated that, while MASLD was exacerbated, these conditions failed to induce hepatic fibrosis, a key component of human MASLD.^[^ ^12^ ^]^ Further research revealed that a 16‐week thermoneutral housing period coupled with a Western diet did not sufficiently drive fatty liver progression in mice.^[^ ^13^ ^]^ Therefore, appropriate diet selection is equally as important as thermoneutral housing to accurately simulate human MASLD conditions in murine models.^[^ ^14^ ^]^

Numerous diets featuring diverse nutrients have been constructed for disease modeling; however, existing models display key shortcomings. For instance, the frequently employed HFD reliably induces common metabolic disorders like obesity, insulin intolerance, and dyslipidemia. Yet, to consistently trigger liver steatosis, an extensive feeding period of at least 20 weeks is necessary, and even then, mild MASH only emerge between 24–60 weeks. To overcome these limitations, many researchers have adopted the methionine‐choline deficient (MCD) diet model. Under this regime, mice exhibit MASH and hepatic fibrosis as early as 6–12 weeks. However, this model does not induce other metabolic disorders such as obesity and thus does not mimic human disease progression, thereby limiting its translational relevance.^[^ ^10^ ^]^ Another key factor that is often overlooked in dietary studies is the crucial role of an appropriate control diet. The frequently utilized diet‐induced models are based on refined ingredient sources. In contrast, many studies still use standard chow diets, derived from complex whole food sources, as controls for comparison. Such comparisons overlook a fundamental factor – the significant impact that the source of diet ingredients can have on gut microbial communities.^[^ ^15^ ^]^ Gut microbiota, with their varied responses to different diet compositions, are increasingly being recognized for their pivotal role in MASLD. The differing impacts on the gut microbiome caused by refined ingredient diets versus complex whole food sources can drastically affect the reproducibility of in vivo experiments. These disparities can yield misleading results, complicating data interpretation. While a meta‐analysis of MASLD models shows that a high‐fat, high‐fructose diet closely mimics human MASLD, these studies mainly compare this to chow diets and depend largely on markers of metabolic syndrome and liver histology scores. Importantly, there are currently no longitudinal studies exploring the impacts of diet‐induced MASLD in murine models, including the physiological and molecular relevance to human disease progression.^[^ ^16^ ^]^ Furthermore, the literature is sparse on studies scrutinizing the impact of nutritional interventions and weight loss strategies on these models. This gap is significant and addressing these shortcomings should be a priority to ensure the development of more effective strategies for managing and treating MASLD.

To progress in MASLD research and treatment, we need a reliable model that accurately reflects the disease's complex and extrahepatic features as seen in human patients. This necessitates a systemic approach that scrutinizes histological changes across key organs, analyzes hepatic transcriptomic trends, and assesses physiological parameters. Moreover, the model should respond to lifestyle changes, aligning with current treatment guidelines for MASLD patients. It is also crucial that the model permits detailed analysis of the disease's early stages before it becomes irreversible. Rapid disease progression within the model is also key to facilitate efficient testing of potential pharmacological and nutritional interventions. Utilizing a purified diet source and a well‐matched control diet in the model is fundamental. This approach reduces batch‐to‐batch variations, ensures better reproducibility, and allows for the precise manipulation of dietary effects. The development of such a preclinical model will catalyze MASLD research, providing an invaluable platform to screen for potential biomarkers and evaluate novel therapeutic approaches.

In this study, we address the gaps in MASLD preclinical modeling by adopting a comprehensive “bedside‐to‐bench and back” approach. We introduce a novel MASLD model, where we undertake detailed longitudinal phenotyping of disease progression, encompassing metabolic parameters, hepatic pathohistology and transcriptomics, and extrahepatic pathology. We identify transcriptomic gene signatures corresponding to key transitory stages of human MASLD, as well as changes in hepatic transcriptome and gastrointestinal metagenome in diet‐induced weight loss. This enables us to demonstrate the clinical relevancy of our model and provides a more dynamic perspective of disease management. Taken together, this systemic biology approach offers fresh insights into MASLD etiology and management, opening new avenues for effective interventions.

## 2 Results

### 2.1 LIDPAD Mice Show Key Progressive Stages of Human MASLD

**Figure 1:**
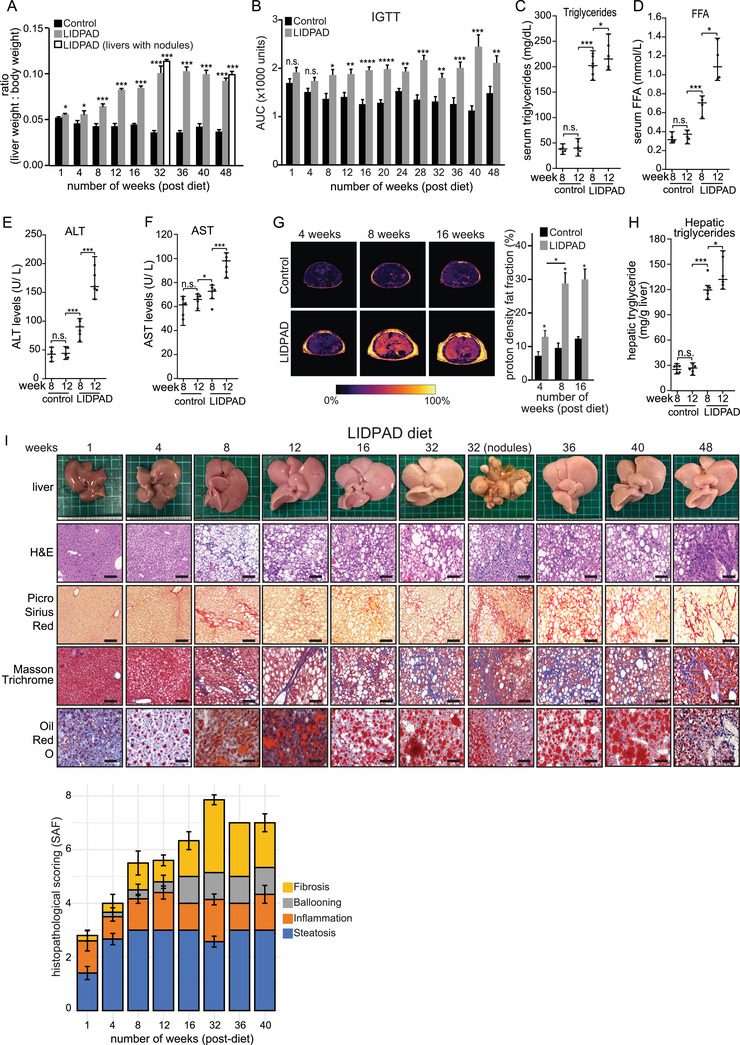
Metabolic parameters of control and LIDPAD mice. A) Liver weight normalized to body weight after feeding for 1 to 48 weeks. B) Area under the curve (AUC) of intraperitoneal glucose tolerance test (IGTT) curves at the indicated timepoints. C,D) Serum triglycerides (C) and free fatty acids (FFA) (D) level in control‐ and LIDPAD‐fed mice at 8 and 12 weeks. The LIDPAD group shows a significant increase in serum triglycerides and FFA compared to the control‐fed mice. E,F) Blood chemistry readout of control and LIDPAD mice based on a liver function test consisting of alanine aminotransferase (ALT) (E) and aspartate aminotransferase (AST) (F). G) Proton density fat fraction (PDFF) images from the liver of 4‐, 8‐ and 16‐week post‐diet intervention control and LIDPAD mice. Increased accumulation of hepatic fat is indicated by the PDFF scale. Quantification of liver PDFF from control and LIDPAD mice at all imaging timepoints (right). Excessive lipid accretion was observed during 8–12 weeks of LIDPAD intervention. Subcutaneous fat growth identified between timepoints. H) Hepatic triglycerides level in control‐ and LIDPAD‐fed mice at 8 and 12 weeks. The LIDPAD group shows a significant increase in serum triglycerides and FFA compared to the control‐fed mice. I) Representative macroscopic and microscopic images of the livers obtained from LIDPAD mice. Histological sections were stained with hematoxylin and eosin (H&E) to show general liver features, picrosirius red, and Masson Trichrome to highlight collagen deposition, and oil red O to detect the presence of lipids. The scale bar represents 100 µm. A bar plot (bottom) shows histological SAF scores of the livers from LIDPAD mice at the indicated weeks post‐feeding. For 1A‐B, *n* = 7–10 for each group. Data are expressed as the means ± SEMs. ****p < 0.0001, ***p < 0.001, **p < 0.01, *p < 0.05 (unpaired *t*‐test, ANOVA Welch's *t*‐test or ANCOVA test when appropriate, followed by post hoc comparisons). n.s. denotes not significant. For 1C‐F, H, *n* = 6 for each group. Data are median ± interquartile range. ***p < 0.001, *p < 0.05 (Mann–Whitney test) For 1G, *n* = 5 for each group, *p < 0.05 (Mann–Whitney test following pixel thresholding at 60%).

Wild‐type C57BL/6J male mice housed in the thermoneutral zone (30 °C) were fed either a high fat‐cholesterol diet of refined ingredients, herein called Liver Disease Progression Aggravation Diet (LIDPAD) mice or a matched Control diet with refined ingredients to ensure comparability (Table S1, Supporting Information). LIDPAD mice exhibited rapid weight gain, both in body and liver, within the first week of their diet, and continued to outpace the control group (**Figure** **1**A; Figure S1A, Supporting Information). Within 8 weeks, these mice developed impaired glucose tolerance, escalating insulin resistance by week 24 (Figure 1B; Figure S1B,C, Supporting Information). By week 8, LIDPAD mice displayed clear signs of hyperlipidemia, with notably increased serum total triglyceride and free fatty acids (Figure 1C,D). Additionally, there was a marked elevation in serum alanine transaminase (ALT) and aspartate aminotransferase (AST) levels from this point, indicating potential liver damage (Figure 1E,F; Figure S1B, Supporting Information).

LIDPAD and control mice underwent magnetic resonance imaging‐based proton density fat fraction of liver at 4‐, 8‐ and 16‐ weeks post‐diet initiation. At all imaging timepoints, LIDPAD mice had a significantly increased liver fat fraction compared with control mice (Figure 1G). Mice on the LIDPAD diet exhibited a steady increase in hepatic proton density fat fraction (PDFF), starting from 12.9% at 4 weeks post‐diet to 30.1% at 16 weeks. Notably, there was a 2.2‐fold increase in liver fat fraction between the 4‐ and 8‐week imaging intervals. From weeks 8 to 16, the rise in hepatic lipid accumulation was more gradual, with PDFF increasing by just 1.3%. In comparison, control mice showed a modest elevation in liver fat fraction over the same periods, with PDFF values ranging from 7.4% to 12.5% (Figure 1G). Both PDFF imaging and total hepatic triglyceride levels (Figure 1H) showed that LIDPAD feeding leads to increased visceral and hepatic adiposity in mice.

Histological liver examination remains the gold standard for diagnosing MASH. Liver sections of LIDPAD and control mice at various weeks of diet were scored using the steatosis, activity, and fibrosis (SAF) system. Hepatic lipid accumulation was evident between 1–4 weeks of LIDPAD feeding but was absent in the livers of control mice (Figure 1I; Figure S2A, Supporting Information), consistent with the MRI imaging data. After 4 weeks, the livers of LIDPAD mice were pale and enlarged with intracellular lipid vacuoles, supported by a SAF score of either grade 2 or 3 in all liver specimens. By 8–12 weeks, mice under LIDPAD developed widespread steatosis, varying levels of intralobular inflammation, and infiltration of lymphocytes and macrophages, similar to what is observed in human MASH. In particular, hepatocyte ballooning, the characteristic feature of MASH was seen by 8 weeks. (Figure 1I; Figure S2B, Supporting Information). Fibrosis was prevalent in 60–80% of the mice. With prolonged feeding of LIDPAD, increased severity of hepatic fibrosis and more extensive lipid accumulation were observed, except for weeks 32 and 48 (Figure 1I; Figure S2B, Supporting Information). These instances of reduced steatosis were likely due to decreased lipid accumulation when fibrosis became more prevalent. Liver specimens from weeks 16 through 40 indicated a sustained and consistent increase in all aspects of steatosis, ballooning, inflammation, and fibrosis (Figure 1I; Figure S2A,B, Supporting Information). From and beyond week 32, hepatocellular nodules were observed in ≈13% of the mice, comparable to the rate of ≈2–12% for human hepatocellular carcinoma.^[^ ^17^ ^]^

Next, we assess the impact of diet composition on MASLD by feeding mice either an HFD (45% calories from fat) or the LIDPAD, while keeping them in a thermoneutral environment for up to 16 weeks. Mice on both diets exhibited significant obesity, evidenced by rapid weight gain compared to the control group (Figure S3A, Supporting Information). The HFD mice developed glucose intolerance as early as one week, whereas this condition emerged after 8 weeks in the LIDPAD group (Figure S3B, Supporting Information). Despite considerable weight gain and insulin resistance, liver histopathology in the HFD group remained mostly unremarkable, showing only mild steatosis and lack of MASH symptoms such as immune infiltration, ballooning, and fibrosis even at 16 weeks (Figure S3C, Supporting Information). After 8 weeks on the HFD, mice exhibited increased levels of circulating and hepatic triglycerides and cholesterols, indicative of hyperlipidemia (Figure S3D,E, Supporting Information). By 12 weeks, these mice also showed elevated AST and ALT levels, markers of liver injury, which coincided with heightened expression of inflammatory genes *IL6, Cxcl10*, and *Ccl20* (Figure S3F–L, Supporting Information). Despite these changes, fibrosis remained marginal at 12 weeks, with only mild increases in the expression of collagen genes, *Col1a1* and *Col4a3* (Figure S3M–O, Supporting Information). In contrast, the LIDPAD diet induced more pronounced metabolic dysregulation, liver damage, and marked increases in inflammatory and fibrotic gene expression by 8 weeks compared to the control and HFD diet groups. This suggests that MASLD progression is notably slower under HFD conditions compared to LIDPAD, highlighting the critical role of diet composition and thermoneutrality in fully manifesting the spectrum of MASLD symptoms.

Altogether, the LIDPAD mouse model developed metabolic dysfunctions and presents the spectrum of MASLD disease progression, with steatosis starting in the early timepoints (weeks 1–4) and MASH appearing after 8 weeks. Fibrosis was noted in 80% of mice starting at 12 weeks. A proportion of LIDPAD mice eventually develop end‐stage sequelae such as cirrhosis and hepatocellular nodules, mirroring MASLD patients in disease progression and incidence.

### 2.2 MASLD‐Associated Extrahepatic Complications Observed in LIDPAD Mice

**Figure 2:**
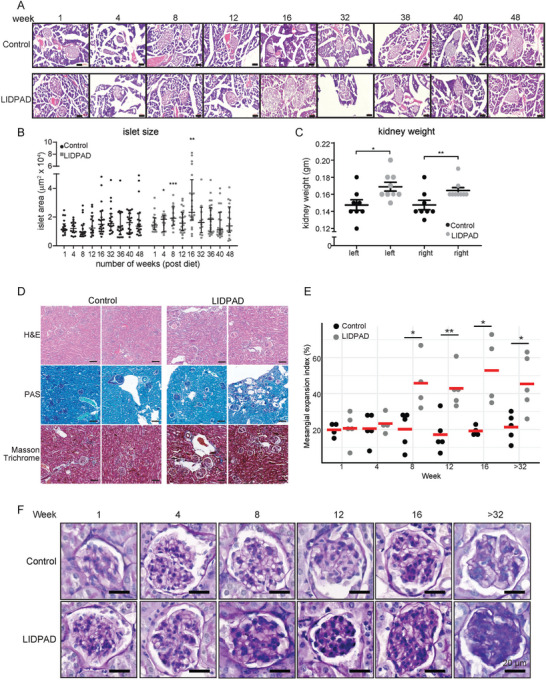
Pancreas and kidney pathology of control and LIDPAD mice. A) Representative H&E images of pancreatic islets. The scale bar represents 100 µm. B) Quantification and analysis of islet area for control and LIDPAD mice across weeks 1 to 48. *n* = 12–28 region of interest (≥5 islets per animal). Data are expressed as the median ± interquartile range. C) Kidney weight (both kidneys) of control and LIDPAD mice compared at week 48. *n* = 8 per group. D) Representative images of kidneys stained using H&E to show the general morphology. Periodic Acid‐Schiff (PAS) staining was used to highlight the basement membrane, and Masson trichrome staining was used to identify collagen deposition. The scale bar represents 100 µm. E) Mesangial expansion index of control and LIDPAD mice at different time points. Red bars indicate the mean of each group. F) Representative images of the kidney glomerulus from control and LIDPAD mice. The scale bar represents 20 µm. Data are expressed as the means ± SEMs, ***p < 0.001, **p < 0.01, *p < 0.05 (Welch's *t*‐test). n.s. denotes not significant.

Next, we undertook a comprehensive phenotyping of the LIDPAD mice, focusing particularly on extrahepatic complications. In LIDPAD mice, there was a significant 1.5‐fold increase in the islet area between weeks 4 and 16, which coincided with the onset of glucose intolerance. This suggests a compensatory islet cell hyperplasia, akin to islet compensation seen in pre‐diabetes (**Figure** **2**A,B). From 40 weeks onward, LIDPAD mice began to show smaller islets (≤0.5 µm^2^ × 10^4^) compared to control mice. This change likely reflects a deterioration of islet mass due to prolonged insulin resistance, indicative of the link between MASLD and the development of type‐2 diabetes. In contrast, the control mice displayed no significant change in islet area from week 1 to 48, maintaining an average islet size of 0.5 µm^2^ × 10^4^ (Figure 2B). Nephromegaly characterized by an enlarged interstitial space, increased immune cell infiltration was also observed in LIDPAD mice at 48 weeks (Figure 2C,D). Kidney sections also showed fibrosis, suggesting prior renal injury. Remarkable mesangial expansion was observed after 8 weeks of LIDPAD feeding, as characterized by significant thickening of the mesangial matrix in the kidney glomeruli (Figure 2E,F). Given the impaired ability of these mice to metabolize glucose, kidney enlargement is likely a result of diabetic nephropathy, although this aspect was not further studied here.

**Figure 3:**
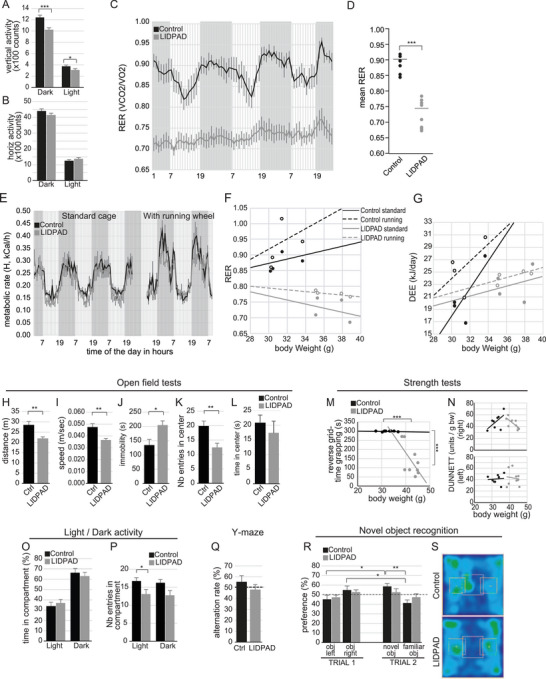
Physiological and behavioral tests of control and LIDPAD mice. A,B) Movement of the animals in terms of vertical (A) and horizontal (B) activity counts recorded over 4 days in the chamber. The light/dark phase allows us to determine circadian changes in the mice during the experiment. C,D) Respiratory exchange ratio (RER) (C) of mice fed the control and LIDPAD diets every hour. Gray and white zones represent dark and light cycles that alternate from 0700 to 1900 daily. Mean RER (D) was calculated to determine any statistical differences between control and LIDPAD mice. E–G) Mouse physiology was recorded when challenged with voluntary exercise. Metabolic rate (E) displayed with gray and white zones representing dark and light cycles. The respiratory exchange ratio (F) of LIDPAD mice remained below 0.8, and the daily energy expenditure (G) of LIDPAD mice remained relatively unchanged after introducing voluntary exercise. H–L) Recorded parameters of control and LIDPAD mice in the open field test. Total distance covered measured in m presented in (H), average speed in m sec^−1^ (I), and time spent stationary in sec (J). Number of entries into the center of the open field indicated in (K). Time spent in the center in sec (L). M,N) Strength tests of control and LIDPAD mice. Reverse grid test grip duration presented in (M) and Dunnett grip strength test for right forelimb (N – top) and left forelimb (N – bottom). O,P) Number of entries made in light/dark box experiment (O). Time spent in light and dark compartment (P). Q) Alternation rate in Y‐ maze. R,S) Time spent on investigating objects in novel object recognition test (R). Trial 1 exposes mice to stimuli for the first time while trial 2 replaces one of the objects with a novel one. Heatmap of total movement in novel object recognition test presented in (S). Data information: *n* = 8 per group. Data expressed as means ± SEM, ***p < 0.001, **p < 0.01, *p < 0.05 (ANOVA Welch's *t*‐test or ANCOVA test when appropriate, followed by post‐hoc comparisons). n.s. denotes not significant.

LIDPAD and control mice were more active during the dark than in the light cycle (**Figure** **3**A,B). While vertical activity was reduced in the LIDPAD mice, a possible result of an increase in their body weight, there was no difference in horizontal activity of both groups (Figure 3A,B). Whole‐body calorimetric analysis revealed that LIDPAD mice has a less obvious circadian oscillation between carbohydrates and lipid utilization with a stable low respiratory exchange ratio (RER 0.70–0.75), indicating a predominant utilization of lipids as fuel compared with control mice (Figure 3C,D). Next, we introduced running wheels to assess the adaptation of the mice to voluntary exercise. Although running wheels increased the metabolic rate in both groups during the dark phase, this increase was relatively muted in the LIPDAD mice compared to controls (Figure 3E). The RER of the control mice oscillated between 0.85 and 1.05 (Figure 3F), suggesting that these mice favored carbohydrates as the primary energy source. In contrast, LIDPAD mice did not adapt to carbohydrate use, although present in a similar percentage kcal as fat in LIDPAD, as evidenced by their RER and daily energy expenditure (Figure 3F,G). These findings indicate that the LIDPAD mice have a disrupted circadian regulation of substrate utilization.

While MASLD itself does not directly cause hepatic encephalopathy (HE), in advanced stages of MASLD, particularly in MASH patients with liver cirrhosis, the risk of developing HE may increase. The severity of HE can range from mild cognitive impairment to severe confusion, coma, and even death. Various human studies suggest that MASLD patients are associated with lower cognitive performance independent of cardiovascular disease and its risk factors.^[^ ^18^ ^]^ Thus, LIDPAD and control mice at 48 weeks of diet were tested in a battery of behavioral assays. Multiple assessments confirmed that LIDPAD mice were less active, as evidenced by a decrease in the total distance covered and speed in the open field test (Figure 3H,I), and a parallel increase in their resting time or immobility (Figure 3J). These mice also displayed impaired strength in the reverse grid test (Figure 3M). However, the difference between both forepaws of LIDPAD and control mice was not significant in the DUNNETT grip strength test (Figure 3N). As motor deficiency and increased anxiety could explain the perceived decrease in athleticism (distance covered and speed), we assessed anxiety and exploratory behavior using the Open field, Light/Dark box, and Y‐Maze tests. LIDPAD mice were more inclined to display anxiety‐like behavior, as shown by their avoidance to enter in the center of the open field arena (Figure 3K). Once at the center, the time spent there remained similar (Figure 3L). The exploratory rate between LIDPAD and control mice did not differ much in the Dark compartment (Figure 3O), while LIDPAD mice made fewer entries into the Light compartment compared to control mice (Figure 3P). Cognitive abilities were tested in the Y‐Maze and with the Novel Object Recognition paradigm in an open field. The difference in the exploratory rates between LIDPAD and control mice in the Y‐maze was not significantly different (Figure 3Q). Last, we investigated the memory capacity of the mice via a novel object recognition test. In trial 1, the two groups of mice showed similar interest to the objects when these objects were first introduced (Figure 3R). In trial 2, where one of the objects was replaced, the control mice were able to differentiate between the novel and familiar objects. However, the LIDPAD mice did not remember the familiar object and again investigated it considerably (Figure 3R,S), suggesting a short‐term memory deficit.

In LIDPAD mice, we observed obesity, glucose insensitivity, increased adiposity, and dyslipidemia, along with pancreas dysfunction and chronic kidney disease. The leading cause of mortality in MASLD patients is cardiovascular complications. Consistent with these clinical observations, LIDPAD mice have also previously been reported to exhibit vasculopathy, which is relevant to the cardiovascular issues seen in MASLD patients.^[^ ^19^ ^]^ These are common metabolic‐associated extrahepatic dysfunctions found in MASLD patients.^[^ ^20^ ^]^

### 2.3 Longitudinal Analysis Reveals Liver Inflammation and Fibrosis through Transcriptomic and Cytokine Profiling

**Figure 4:**
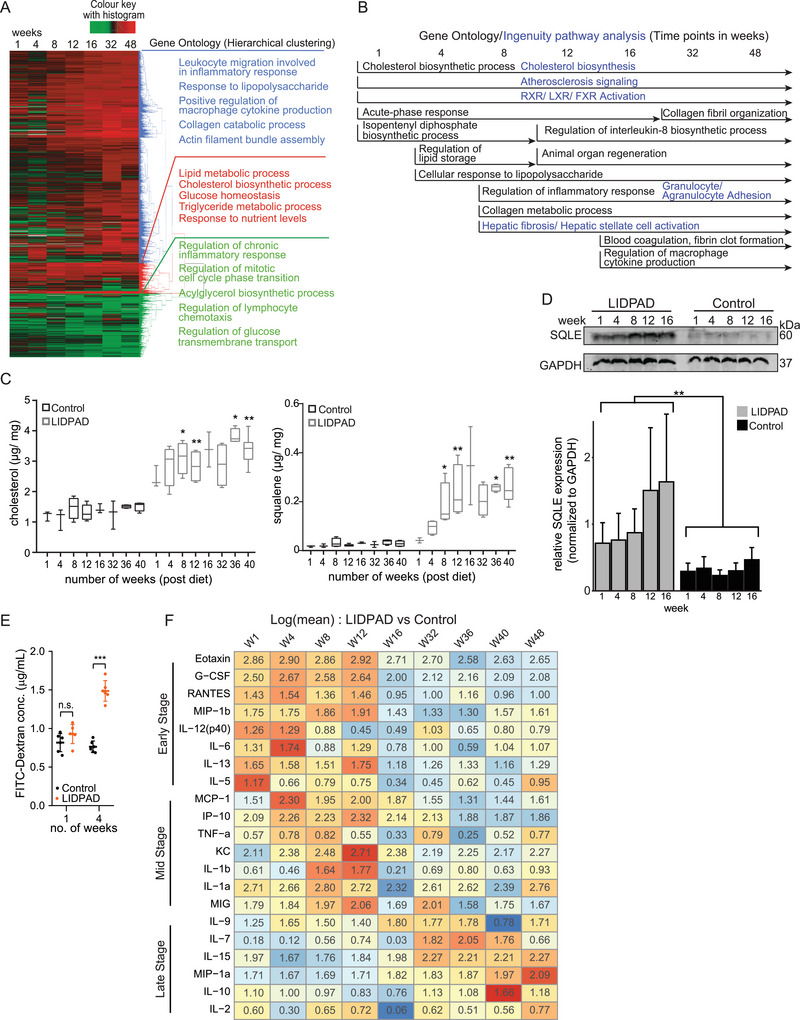
Hepatic transcriptomic profiling of control and LIDPAD mice. A) Heatmap of differentially expressed genes (DEGs) across weeks 1 to 48 with corresponding Gene Ontology (GO) biological process terms guided by hierarchical clustering. B) Gene ontology and pathway analysis across multiple time points tracking temporal changes in DEGs, featuring only recurring GO terms that span across at least two time points, to increase the confidence of the data as a true positive. C) Lipid analysis highlighting cholesterol (left) and squalene (right) differences between control and LIDPAD mice. D) Representative western blot for squalene epoxidase (SQLE) from of livers of indicated groups at various time points. GADPH, as loading and transfer control, was from the same samples. **p < 0.01 (Mann–Whitney *u*‐test). E) Plasma concentration of FITC‐dextran in mice fed with control and LIDPAD diets for 1‐ and 4 weeks after FITC‐dextran gavage, indicating intestinal permeability. *n* = 6 mice per group. ***p < 0.001 (Mann–Whitney u test). n.s. denotes not significant. F) Heatmap of the serum cytokine array with color based on the row values after LIDPAD feeding for 1 to 48 weeks. Data are expressed as log (fold change of mean concentration) between LIDPAD and control mice.

To gain a comprehensive understanding of the mechanisms driving the observed histological changes in MASLD, we conducted thorough transcriptomic and biochemical analyses. By applying hierarchical clustering to the differentially expressed genes (DEGs) obtained from liver samples of LIDPAD mice, we successfully identified three distinct gene groups characterized by diverse Gene Ontology profiles (**Figure** **4**A,B). Among the key early events, the dysregulation of cholesterol and lipid homeostasis stood out, along with the activation of the acute‐phase response. These observations strongly indicate that hepatic inflammation is a consequence of lipotoxicity. To corroborate these findings, we conducted liquid chromatography‐mass spectroscopy analysis, which showed elevated levels of free cholesterol and squalene in LIDPAD mice compared to the control group (Figure 4C). The livers of control mice showed a basal level of cholesterol, consistent with normal cholesterol synthesis, and minimal squalene levels (Figure 4C). The increase in free cholesterol likely stems from its presence in the LIDPAD diet, while the rise in squalene concentration suggests an alteration in the sterol biosynthetic pathway caused by the LIDPAD diet. A key enzyme in this pathway, squalene epoxidase (SQLE), which converts squalene to squalene to (S)−2,3‐epoxysqualene, was also found to be elevated in liver samples from LIDPAD mice (Figure 4D). Disruption of SQLE function is linked to cholesterol metabolism disorders and has been implicated in various diseases, including MASLD. Our analysis revealed a significant shift in the hepatic transcriptomic profile in LIDPAD mice, especially in their cellular response to lipopolysaccharide (LPS) within weeks 1–4 (Figure 4A,B). This finding indicates a compromised gut barrier integrity, supported by a marked increase in gut permeability (Figure 4E), a factor contributing to the development of MASLD. As MASLD progressed, there was a notable rise and intensification in the expression of genes associated with collagen assembly and inflammatory response, corroborating our histological and pathological findings.

Several studies have highlighted the significance of serum cytokines in the assessment of MASLD patients.^[^ ^21^ ^]^ Through multiplex immunoanalysis of serum samples, we made an intriguing observation: no single cytokine remained consistently elevated throughout the entire progression from steatosis to cirrhosis. Instead, we identified distinct yet overlapping early‐, mid‐, and late‐stages of cytokine profiles, providing valuable insights into the disease dynamics (Figure 4F). During weeks 1 to 4, we noticed an upregulation of low‐grade chronic inflammatory cytokines (IL‐6 and IL‐13) and inflammatory chemokines (MIP‐1α and eotaxin), which concurred with the onset of steatosis. Moving to weeks 8–12, the inflammatory response was sustained by a new group of cytokines (TNF‐*α* and IL‐1*α*) and chemokines (MCP‐1 and MIG) that further promoted the infiltration of immune cells, exacerbating the inflammatory process. Beyond 12‐week, there was a decline in the early‐ and mid‐stage cytokines, with subsequent emergence of a different group of cytokines crucial for the maturation of adaptive immune cells (IL‐7, IL‐9, IL‐10, and IL‐2) (Figure 4F). This suggests a transition in the immune response as the disease progresses. Our findings emphasize the importance of using varied panels of cytokines/chemokines to effectively monitor the evolving stages of MASLD.

Our comprehensive transcriptomic analysis and cytokine profiling have provided critical insights into the cellular mechanisms and inflammatory responses driving MASLD progression. These findings highlight the complex interplay between cellular processes and mechanisms and inflammation in disease development, reinforcing the need to include a diverse set of biomarkers to monitor disease progression effectively.

### 2.4 Comprehensive Staging of MASLD in the LIDPAD Mice using Human Transcriptomic‐Guided Analysis

**Figure 5:**
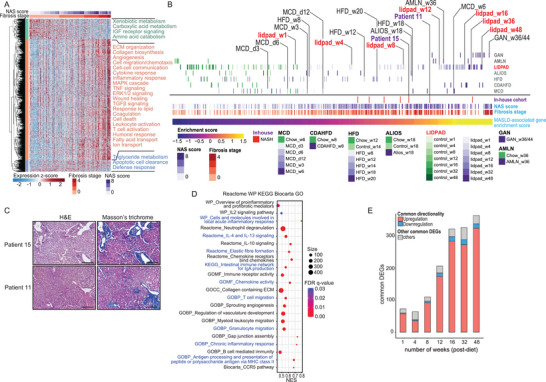
Human transcriptomic‐guided staging of LIDPAD model. A) Heatmap of 1198 hepatic DEGs associated with NAS score and fibrosis stage in MASLD patients obtained from public clinical cohorts. The DEGs were hierarchically clustered into three functional groups based on their expression pattern and the associated biological functions are annotated. B) Transcriptomic‐guided staging of LIDPAD models and other diet‐induced MASLD mouse models based on ranked enrichment scores of a unified MASLD transcriptomic signature comprised of 54 genes. The MASLD transcriptomic signature was established based on the patient cohorts in A and the respective NAS scores and fibrosis stages of each individual are annotated. The enrichment scores of our in‐house MASH patients and different diet‐induced MASLD models at varying feeding durations (d: days; w: weeks) are also included. ALIOS, American lifestyle‐induced obesity syndrome diet; AMLN, Amylin liver MASH diet; CDAHFD, choline‐deficient, *L*‐amino acid‐defined, high‐fat diet; GAN, Gubra‐Amylin MASH diet; HFD, high‐fat diet; LIDPAD, Liver Disease Progression Aggravation Diet; MCD, methionine choline‐deficient diet. C) Representative haematoxylin & eosin (H&E) and Masson's trichrome images of the liver biopsies from our in‐house MASH patient cohort. Scale bar: 200 µm. D) GSEA plotted using FDR and size, showing enriched terms from Reactome, WP, KEGG, Biocarta, and GO in the liver transcriptomes of MASH patients (*n* = 10) compared to normal liver tissues (*n* = 5) from our in‐house patient cohort obtained from the Fifth Affiliated Hospital of Sun Yat‐Sen University. E) Number of common DEGs between the transcriptomic profiles of LIDPAD mice at different time points and human DEGs associated with MASLD progression. Red and blue bars denote DEGs that are consistently up and downregulated in both human and mouse MASLD. Grey bars represent the common DEGs that showed inverse expression trends between humans and mice MASLD but only make up a small portion of the total common DEGs.

We validated the clinical relevance of our model by establishing MASLD‐associated transcriptomic signatures using liver transcriptomes of MASLD patients from gene expression omnibus (GEO) (Figure S4A, Supporting Information). Four patient liver RNAseq datasets (GSE130970, GSE162694, GSE207310, GS225740; *n* = 306) with varying MASLD Activity Score (NAS) and fibrosis stages were included. We performed a meta‐analysis with batch and gender correction to integrate the datasets and revealed a gradual shift of the liver transcriptomes according to MASLD severity (Figure S4B, Supporting Information). Differential expression analysis identified three functional clusters of DEGs (a total 1198 genes) associated with NAS and fibrosis stages (**Figure** **5**A, Data 1, Supporting Information). One cluster consisted of genes that were downregulated as the disease advanced, primarily involved in xenobiotic metabolism, amino acid catabolism, and insulin‐like growth factor receptor signaling. The middle, and largest, cluster comprised genes that were overexpressed in MASLD and played roles in inflammatory and immune responses, lipid transport and metabolism, fibrogenesis, pro‐coagulation, and cell death. The last cluster, associated with triglyceride metabolism, apoptotic cell clearance, and defense response, showed upregulation in the early stage of MASLD, but its expression subsided in the late fibrotic stage.

However, utilizing the 1198 human MASLD‐associated DEGs for transcriptomic‐guided staging can be computationally demanding and time‐consuming. To address this, we employed the Boruta feature selection algorithm, which is based on the Random Forest Classifier, to identify key gene signatures.^[^ ^22^ ^]^ This process resulted in 89 key gene features. We further refined the selection by excluding genes inversely associated with MASLD progression (i.e., downregulated as the disease worsens) and genes without a mouse ortholog, yielding a unified MASLD transcriptomic signature comprising 54 genes (Figure S4C, Data 2, Supporting Information). To assess disease severity, we utilized a single‐sample gene set enrichment analysis (ssGSEA) with the 54‐gene signatures. Analysis of the integrated MASLD patient cohorts demonstrated a strong positive Spearman correlation between the gene set enrichment scores and NAS score (Spearman's rank correlation *ρ *= 0.6905, *p*‐value < 0.001) as well as fibrosis stage (Spearman's rank correlation *ρ* = 0.6597, *p*‐value < 0.001) of the patients (Figure S4D, Supporting Information).

To validate the robustness of our approach, we applied the pipeline to a validation dataset consisting of six human MASLD hepatic transcriptomic studies (GSE115193, GSE126848, GSE147304, GSE160016, GSE174478, and GSE175448; *n* = 206). Although the metadata of these transcriptomic data included disease staging but not histological scoring, the enrichment scores of the MASLD transcriptomic signatures successfully stratified the patients according to their clinical staging, despite the significant overlap between healthy and MASL individuals, likely due to mild symptoms (Figure S4D,E, Supporting Information). Additionally, our in‐house human MASH liver transcriptomes closely clustered with clinical MASLD samples exhibiting NAS > 5 and fibrosis stage > 2 (Figure 5B). Histological examination of liver specimens from in‐house dataset showed similar concordance when using ordinal SAF staging and enrichment with a unified gene signature (Figure 5C). Further, our GSEA underscored the enrichment of inflammatory and fibrogenic processes in these patients (Figure 5D), corroborating previous findings. Taken together, the matching transcriptomic profiling from both the validation dataset and our in‐house cohort, and the aligned histological analysis of in‐house samples demonstrates the applicability and robustness of our methodology for staging MASLD in newly acquired liver transcriptomes.

Our analytical pipeline allowed us to extend the transcriptomic‐guided staging to various diet‐induced MASLD mouse models (Figure 5B). Our results revealed that diets such as the MCD diet or the choline‐deficient, *L*‐amino acid‐defined, high‐fat diet (CDAHFD) could induce severe fibrosis (F3‐4) within 6 to 8 weeks but failed to mimic human fatty liver and MASH effectively. On the other hand, the American lifestyle‐induced obesity syndrome (ALIOS) diet and high‐fat diet (HFD) only achieved mild MASH after 18 to 20 weeks of feeding. In contrast, LIDPAD mice demonstrated the ability to model the full disease spectrum of MASLD within a relatively short time frame. Feeding the LIDPAD diet for 4 weeks resulted in MASLD with a severity comparable to 12 weeks of HFD. Eight weeks of the LIDPAD diet exhibited a similar disease progression to 18 weeks of HFD and American Lifestyle‐Induced Obesity Syndrome (ALIOS), representing the early stages of human MASH. Furthermore, 12 weeks of the LIDPAD diet induced severe fibrosis equivalent to six weeks of MCD and 36 weeks of the Amylin liver MASH (AMLN) diet. Prolonged feeding with LIDPAD (16 weeks onward) successfully replicated late‐stage human MASH and cirrhotic livers, which are typically achieved only after extended feeding periods of 36 to 44 weeks with the Gubra‐Amylin MASH (GAN) diet or 8 weeks of CDAHFD, the latter of which is specialized for liver fibrosis rather than MASLD. Comparing the temporal gene expression patterns in our LIDPAD models with the human MASLD‐associated DEGs revealed a steady increase in the overlap of DEGs with prolonged LIDPAD feeding (Figure 5E). This finding confirmed the relevance of the LIDPAD model in recapitulating key molecular features of human MASLD. Notably, we observed a sharp surge in the overlapping DEGs between weeks 4 and 16, indicating a rapid transition from simple steatosis to MASH with intensifying fibrotic severity within 4 months.

In summary, we have successfully established a unified gene signature and an analytical pipeline for transcriptomic‐guided staging of MASLD in both humans and mice. Through this molecular approach, we demonstrated that the LIDPAD model closely resembles human MASLD pathophysiology and enables efficient modeling of disease progression within a relatively short time frame of 4 to 16 weeks.

### 2.5 LIDPAD Mice Respond to Diet‐Induced Weight Loss and Reduce MASLD Severity

**Figure 6:**
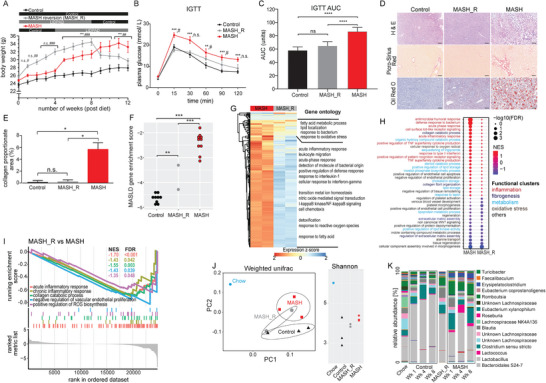
Diet‐induced weight loss on MASH progression. A) Weight of LIDPAD mice at 8 weeks to achieve MASH and then fed control diet for a further 4 weeks. Data expressed as mean ± SEM ***p < 0.001, **p < 0.01, *p < 0.05 (Kruskal–Wallis test with Dunn test for multiple comparisons). n.s. denotes not significant. B,C) Intraperitoneal glucose tolerance test. B) IGTT curves and C) AUC of control, MASH mice that underwent dietary reversion, and MASH (8 weeks) mice. Data information: *n* = 10 per group (body weight), 5 per group (IGTT). Data expressed as mean ± SEM ****p < 0.0001, ***p < 0.001, **p < 0.01, *p < 0.05 for comparison of LIDPAD versus control groups, ### *p *< 0.001, ## *p *< 0.01, # *p *< 0.05 for comparison of reversion versus control groups (Mann–Whitney *u*‐test). n.s. denotes not significant. D) Representative images of liver sections obtained from diet reversion of MASH. The scale bar represents 100 µm. *n* = 3–4 mice per group. E) Bar graphs denote collagen proportionate area (CPA) calculated from PSR staining of liver sections obtained from phenotypic reversion of MAFL (E), and MASH (F). *n* = 3–4 mice per group. Data expressed as mean ± SEM ***p < 0.001, **p < 0.01, *p < 0.05 (Kruskal–Wallis test with Dunn test for multiple comparison). n.s. denotes not significant. F) Enrichment scores of the unified MASLD transcriptomic gene signature of control, MASH mice with dietary reversion, and MASH mice. ***p < 0.001, **p < 0.01 (one‐way ANOVA followed by post hoc pairwise comparisons with FDR adjustment). G) Heatmap of DEGs with Gene Ontology performed on clusters defined by hierarchical clustering. Transcriptomic profiles of mice fed with MASH and MASH_R were visualized against control samples as the basis of comparison. H) GSEA analysis of MASH and MASH_R compared against control samples respectively. I) Enrichment plots of MASH_R versus MASH hepatic transcriptomic profiles in gene sets related to acute inflammation, chronic inflammation, collagen catabolism, suppression of vascular endothelial proliferation, and ROS biosynthesis. J) Bacterial *β*‐diversity (weighted UniFrac, left) and *α*‐diversity (Shannon index, right) of the stool samples from mice on different feeding regimens. K) Relative abundance of bacteria genera in stool samples of control, MASH, and MASH_R mice. Only bacteria genera that constitute more than 1% of the abundance are annotated.

Lifestyle modifications aimed at weight loss have been shown to be an effective management strategy for patients with MASLD. To investigate the effects of weight loss through diet reversion, LIDPAD mice were initially fed the LIDPAD diet for 8 weeks to induce MASH. Subsequently, they were switched to the control diet (MASH_R) for 4 weeks to simulate short‐term dietary counseling in MASLD patients. Remarkably, mice in the reverted MASH_R groups experienced 14% weight loss compared to those that continued the LIDPAD diet over the same period (**Figure** **6**A). This weight loss in the reverted MASH_R group not only improved glucose tolerance (Figure 6B,C) but also led to significant improvements in liver histology and collagen proportionate area, with minimal hepatocyte injury, reduced lipid accumulation, and the absence of fibrosis (Figure 6D,E). Furthermore, liver histopathological changes were reflected in the gene signature of the MASH_R mice, revealing insights into the effects of diet on the hepatic transcriptome. The transcriptomic profiles of these mice were distinctly different, with key disease‐associated signatures reversing upon dietary reversion (Figure 6F). Analysis of DEGs in the MASH_R mice showed a marked downregulation in several processes, such as inflammation, immune response, oxidative processes, and collagen assembly (Figure 6G, Data 3, Supporting Information). Confirming this observation, the GSEA of MASH and MASH_R genes compared to the control group, showed the mitigation of MASH‐related hepatic injuries such as inflammation, fibrogenesis, lipid dysmetabolism, and oxidative stress (Figure 6H). Intriguingly, while diet reversion did not trigger a liver regenerative response, as evidenced by the suppression of genes associated with tissue remodeling and regeneration, we observed significant enrichment of repair mechanisms involving endothelial cells. This was marked by a decrease in genes promoting endothelial cell apoptosis and those inhibiting endothelial proliferation, along with an increase in axon regeneration in the liver neuronal network (Figure 6H,I). The liver neuronal network has been found to have pathways that travel to other organs like adipose tissues, modulating systemic glucose and energy metabolism. A direct comparison MASH_R and MASH transcriptomes further confirms that dietary reversion effectively attenuates the transcriptional regulation of disease features such as hepatic fibrosis, inflammation, lipid dysmetabolism, suggesting a potential for liver repair in vascular layers (Figure 6I). This data offers valuable insights into the mechanisms by which diet interventions can reverse MASH.

The gut microbiome plays a crucial role in modulating host metabolism and immune function, and its composition can be influenced by dietary factors. By analyzing the gut microbiome alongside DEG analysis, we can gain a comprehensive understanding of the interplay between diet, gene expression, and microbial ecology, contributing to our understanding of how dietary interventions impact liver health in MASLD. Our findings showed notable differences in the gut microbiome of mice fed a grain‐based chow diet compared with those on the control and LIDPAD diets with refined ingredients. Weighted UniFrac analysis for *β*‐diversity revealed distinct clustering of mice on control or LIDPAD diets, with those on diet reversion (MASH_R), occupying an intermediate position between these two groups (Figure 6J,K). The gut microbiome of mice on the chow diet exhibited high Shannon *α*‐diversity, indicating a more diverse microbial composition. In contrast, mice on the carbohydrate‐enriched control diet displayed a reduction in the Shannon *α*‐diversity index by week 8–12, pointing to changes in microbial diversity. Interestingly, the gut microbiome of LIDPAD mice remained relatively stable throughout the feeding period. In the MASH_R group, the *α‐*diversity was intermediate between the control and LIDPAD mice at the same time point (Figure 6J). Upon closer examination, we found that genera such as *Lachnospiraceae*, which were enriched in the chow diet, exhibited lower abundance in the control diet promoting the growth of *Lactobacillus* (Figure 6K). Furthermore, LIDPAD‐fed mice showed a higher abundance of *Erysipelatoclostridium*, *Romboutsia*, and *Lactococcus*. However, in the mice subjected to diet reversion, the relative abundance of these bacterial genera was intermediate between that of the LIDPAD and control diets, with a notable reduction in three LIDPAD‐enriched genera. These observations underscore the influence of diet on the gut microbiome and emphasize the importance of maintaining a stable gut microbial ecology for liver health.

Our findings from the LIDPAD model affirm that dietary intervention promoting weight loss can positively influence MASH prognosis, as supported by histological examination and changes in hepatic transcriptomic profiles. Importantly, our study underscores the impact of diet on gut microbiome composition, suggesting that a stable gut microbial ecology is crucial for improving liver health. These findings reinforce current dietary recommendations for MASLD patients and expand our understanding of the role of gut microbiome in liver disease. This knowledge supports the development of precise dietary strategies tailored to manage MASLD effectively.

## 3 Discussion

The recognition of MASLD as a disorder with effects extending beyond the liver underscores the importance of developing models that accurately reflect its extrahepatic complications.^[^ ^23^ ^]^ Our study introduces a diet‐induced MASLD mouse model using the LIDPAD, specifically to replicate the multifaceted nature and comorbidities of human MASH. This model addresses the inadequacies of current preclinical approaches, which could be a factor in the stalled development of MASLD therapies. By employing thermoneutral housing to align more closely with human physiology,^[^ ^24^ ^]^ and using precision dietetics to avoid inconsistencies in diet composition which are metabolic confounders,^[^ ^15^, ^25^ ^]^ the LIDPAD model represents a significant advance in MASLD research, bridging gaps and pioneering new directions in the field.

The LIDPAD MASLD model stands out for its rapid disease progression and accurate replication of the diverse features of human MASLD, including anthropometric, physiological, histological, and transcriptomic aspects. This model allows for an expedited and in‐depth disease analysis. LIDPAD mice rapidly gain weight and exhibit impaired glucose homeostasis, paralleling human disease progression. Our approach, encompassing non‐invasive imaging, liver histology, and hepatic transcriptomics over time, revealed that LIDPAD mice develop steatosis within 1–4 weeks, inflammation by 4–8 weeks, and escalating fibrosis from 12–16 weeks. Serum profiling in LIDPAD mice uncovers stage‐specific cytokine trends, offering new insights into biomarkers for tracking human MASLD progression, a step beyond conventional static disease models. This is a major advancement over existing preclinical MASLD studies that often use separate models for different stages of the disease, failing to emulate the dynamic progression of the human condition. Unlike HFD models that quickly develop steatosis and glucose intolerance but rarely progress to significant fibrosis, even after extended periods, the LIDPAD model closely aligns with the human MASLD trajectory. It overcomes limitations of other diets, like MCD, which fail to fully capture the complex diabetes‐obesity interplay in human MASLD, often leading to skewed interpretations.

Unique to our study is the use of temporal transcriptomic staging in LIDPAD mice, a methodology that provides deeper insights than traditional histological staging.^[^ ^26^ ^]^ We first identified the salient transcriptomic features that correspond to key histological and pathological stages in humans. By pooling public datasets of temporal transcriptomes from various diet‐induced models, we found that other models poorly align with human MASLD progression. Our approach emphasizes transcriptomic features that align with key MASLD stages in humans, distinguishing the LIDPAD model. It not only replicates human disease progression but also shows high relevance, evidenced by a significant percentage of DEGs that correlate with findings in human studies. This method enhances analytical precision and staging capabilities, surpassing the resolution offered by traditional histological staging, establishing the LIDPAD model as a highly relevant tool for human disease research.

The complexity of MASLD, driven by complex gene‐environment interactions and multi‐organ communication such as the gut‐liver and gut‐pancreas axes, often manifests in extrahepatic conditions including insulin resistance, cardiovascular complications, and changes in gut microbiota. Past research on MASLD models typically examined these extrahepatic changes only at study completion, overlooking the critical timeline for conditions like pancreatic expansion and nephromegaly. This oversight limits our understanding of early development and progression of these complications, and importantly, the opportunity for early intervention.^[^ ^27^ ^]^ While the impact of ongoing nutritional stress on the kidney and pancreas is typically studied using an HDF‐induced model, our LIDPAD model effectively traces the early onset and development of multi‐organ dysfunctions, including pancreatic issues, nephromegaly, and shifts in gut microbiota. Our temporal assessment strategy provides insight into MASLD etiology, particularly highlighting crucial early events such as increased gut permeability observed as soon as 4 weeks into the condition. LIDPAD mice show MASLD‐associated vasculopathy characterized by elevated endothelial expression of CXCL12 in the aortas and liver vasculature, similar to human observations.^[^ ^19^ ^]^ This model also uncovers a liver‐pancreas axis that triggers compensatory pancreatic islet cell hyperplasia, a phenomenon observed in animal models of insulin resistance^[^ ^28^ ^]^ and in humans, where a clear correlation exists between body‐mass index, *β*‐cell mass, and the onset of type‐2 diabetes.^[^ ^29^ ^]^ Furthermore, the presence of nephromegaly and fibrotic lesions in LIDPAD mice highlights the importance of investigating the gut‐liver‐kidney axis further. Such insights are vital for identifying early intervention opportunities, providing a strategic edge in addressing the disease at its nascent stages.

A notable finding from our LIDPAD model is the onset of motor neuron dysfunction and cognitive impairments after 48‐weeks of the LIDPAD diet. These symptoms, including reduced forepaw grip strength, decreased physical activity, increased anxiety, and short‐term memory loss, represent serious central nervous system (CNS) complications in advanced MASLD that can range from mild cognitive issues to severe confusion and, in extreme cases, death. The effects of MASLD‐inducing diets on the CNS have been under‐recognized, likely due to the slow disease progression not readily leading to neurological changes. Although HFD impacts on memory are primarily documented in transgenic Alzheimer's disease models (3xTgAD) or aged rats,^[^ ^30^ ^]^ our study, through detailed behavioral assessments, introduces a model of diet‐induced cognitive impairment associated with hepatic dysfunction. This model lays the groundwork for future investigations into the underlying mechanisms.

In managing MASLD, achieving 7–10% weight loss through healthier dietary choices is crucial, as it leads to significant histological improvements, fibrosis regression, and steatosis resolution.^[^ ^6^, ^31^ ^]^ Similarly, LIDPAD mice responded positively to diet‐induced weight loss, exhibiting enhanced glucose tolerance and fibrosis resolution. Yet, the real‐world challenge lies in patient adherence to lifestyle modifications.^[^ ^32^ ^]^ Consequently, studying the molecular mechanisms behind dietary intervention in the LIDPAD model offers a pathway to develop strategies that restore metabolic health. Through the study of DEGs, LIDPAD mice revealed the critical role of transcriptional regulation in the progression of MASLD. Notably, these mice show the capacity to reverse MASH‐related gene expressions through dietary changes. This dietary reversion led to a significant decrease in fibrogenic, inflammatory, and dysmetabolic activities, accompanied by an upregulation of genes linked to vascular and neuronal repair. These findings indicate that reducing dietary stressors can activate the body's repair mechanisms, potentially reversing hepatic damage. This understanding opens new avenues for creating pharmacological interventions that complement dietary modifications, offering a dual approach to treatment. The LIDPAD model serves as a valuable tool for exploring the molecular layers of MASLD, identifying key elements that are often elusive in clinical settings. Its insights into nutri‐transcriptomic adaptability and the reversal potential of diet‐related genetic changes are instrumental in advancing our understanding of MASLD and shaping comprehensive treatment approaches.

The role of gut microbiota as a key factor in MASLD pathogenesis is increasingly recognized.^[^ ^33^ ^]^ Our study, while not directly focused on this aspect, emphasizes the necessity of a well‐defined control diet to ensure accurate comparisons and insights into disease pathology, emphasizing precision in dietetics. Traditional metabolic studies often use standard chow diets that contain unrefined ingredients like wheat, cereals, and animal by‐products as control diets.^[^ ^15^, ^34^ ^]^ However, such diets are prone to variability due to supply chain differences, harvesting conditions, and processing methods, particularly affecting fiber content.^[^ ^35^ ^]^ This variability introduced inconsistencies, potentially confounding experimental results.^[^ ^10^, ^36^ ^]^ In MASLD research, where the gut‐liver axis is pvitol, the dietary fiber content significantly influences the gut microbiota.^[^ ^33^, ^37^ ^]^ Standard chow typically contains 10–20% total fiber, both soluble and insoluble, in contrast to most purified diets which have ≈5% fiber, mainly insoluble cellulose. Soluble fibers are fermentable by gut bacteria, whereas insoluble fibers are not. Given that the liver receives a substantial portion of its blood supply from the splanchnic circulation, it is particularly susceptible to gut‐derived metabolites, making the type and composition of dietary fiber important MASLD research.^[^ ^38^ ^]^ Indeed, our study reveals that mice fed a standard chow diet exhibit higher *α*‐diversity in their gut microbiome compared to those on refined ingredient control diets. This difference highlights the impact of diet composition on gut microbiota diversity, which in turn affects the metabolic and redox capacities of the microbial community.^[^ ^39^ ^]^ Our findings also show a reversal of the MASH‐associated gut microbiome toward a profile akin to the control diet, indicating the significant role of the microbial ecosystem's interactome. This observation reinforces the potential of the LIDPAD and control diets in exploring the gut‐liver axis and its implications, opening possibilities for the development of microbiota‐based live biotherapeutics.

The integration of “bench‐to‐bedside and back” transcriptomic‐guided staging in patients with biopsy‐confirmed MASH, along with the application of the LIDPAD mouse model to mirror human MASLD, represents a transformative approach for deciphering the multisystem pathogenesis of MASLD. This model facilitates the testing of novel therapeutic targets and deepens our understanding of the liver‐inter‐organ axes. Its comprehensive nature promises for advancing the development of impactful therapeutic strategies and interventions aimed at restoring metabolic health, ultimately enhancing patient outcomes.

## 4 Experimental Section

### Animals, Feeding Regimens, and Treatment

Male wild‐type C57BL/6J (Invivos, Singapore) mice aged between 8–9 weeks were given ad libitum access to LIDPAD (Liver Disease Progression Aggravation Diet, modified from Teklad diet TD. 88 137, Envigo, USA; PD23081401, Syse bio, China), high‐fat diet (HFD, 45 kcal% fat, D12451, Syse Bio, China) or a control diet (Teklad Custom Diet, modified from AIN‐93 M, Envigo, USA; PD23081404, Syse Bio, China) (Table S1, Supporting Information) and water. Animals were kept in standard housing cages for up to 48 weeks at a controlled temperature and humidity of 30 °C (thermoneutral zone) and 49.9%, respectively, with a 12‐hour dark‐light cycle. Body weight and food consumption were measured weekly. In the diet reversion experiment, mice were randomly assigned into 3 groups: MASH (8 weeks LIDPAD), MASH_R (8 weeks LIDPAD followed by control diet), and control. The MASH group was given a control diet for 4 weeks and switched to LIDPAD thereafter, while MASH_R was given LIDPAD for 8 weeks and switched to a control diet for the last 4 weeks. The control group was given a control diet for 12 weeks, and the diet for all groups was given ad libitum. The timeframe chosen to induce MASH was based on histology and transcriptomic staging from earlier experiments. Animals were euthanized using CO_2_ prior to blood and tissue collection. Organs were fixed with 4% PFA or snap frozen with liquid nitrogen. All experiments were carried out following the guidelines of the Institutional Animal Care and Use Committee (IACUC) (SingHealth IACUC: #2014/SHS/1008; NTU‐IACUC: A18031, A10032, A18033, A18042, A20055).

### Patient Samples

Ten MASLD patients and 5 liver cancer patients scheduled for liver surgery in the Fifth Affiliated Hospital of Sun Yat‐Sen University were recruited to provide surplus liver tissue excised from the surgery. The patients had no prior or existing liver infection or other active infectious diseases. MASLD liver tissue samples were obtained from the MASLD regions of the 10 MASLD patient livers. Normal liver tissue samples were obtained from the normal tissue surrounding the liver tumors of the 5 liver cancer patients. The obtained samples were then cut in halves and kept either in formaldehyde for histological analysis or in a −80 °C freezer before RNA extraction. The study was approved by the Institutional Review Board of the Fifth Affiliated Hospital of Sun Yat‐Sen University, Zhuhai, China (Approval No. L136‐1), and written consent was obtained from all the participants.

### Behavioral Experiments

A cohort of 18 mice were tested in a battery of behavioral tests. The temperature of the room was constant at 30 °C. Mice were exposed to a battery of tests assessing depression and anxiety‐like behaviors, locomotion, and memory abilities. A minimum of 24 h separated one test from another and tests were performed as possible from 9 am to 12 pm, in a period not longer than 3 h. All tests were video‐recorded, and phenotypes were automatically analyzed by means of ANY‐maze video‐tracking software (Stoelting, IL, U.S.A.) unless otherwise stated.

### Elevated Plus Maze

Mice were put in the central platform of an elevated plus maze (35(L) × 5(W) × 15(H) cm, grey non‐reflective metallic texture, Ugo Basile, Italy) and were allowed to explore freely during 5 min. The room was brightly illuminated.

### Light‐Dark Box

Mice were placed in the center of the light compartment of the Light‐Dark Box (44(L) × 21(W) × 21(H) cm consisting of 2 compartments separated by a small open door – one totally white and open, and the second black with a lightproof black lid) and were allowed to explore freely the maze for 10 min. The maze was brightly illuminated to increase the sensation of anxiety in the light compartment.

### Y‐Maze

Mice were placed in one arm of the Y maze (grey PVC structure in Y shape, each arm measuring 32(L) × 8(w) × 15(h) cm), facing the wall and allowed to freely explore the maze during 5 min. The percentage of alternation was calculated as (number of alternations)/((total number of triads) × 100), where “number of triads” = (total number of arm entries −2), and the “number of alternations” = triad where all 3 arms are represented.

### Open Field and Novel Object Recognition

Mice were placed in the center of an open field (grey PVC 40(L) × 40(W) × 50(H) cm) and allowed to explore the test freely for 10 min. Twenty‐four hours later, the mice were put in the same enclosure in the presence of two similar objects placed at a specific location in the arena. After 10 min of exploration, the mice were put back in their home cage for 30 min before being exposed in the same arena to 2 new objects, one exactly as the two first objects (familiar object) and another different in shape and texture (novel object).

### Reverse Grid

Mice were placed in the middle of a grid (0.8 × 0.8 cm) that is slowly reverted so that the mouse must grip with its 4 limbs to avoid falling. The latency to fall was recorded with a cut‐off time of 5 min.

### Dunnett Grip Strength Test

Mice were tested in the DUNNETT original apparatus 5 times at 2 min intervals and the mean of the 3 last values was taken for statistical analysis. Left and right forelimbs were recorded separately by the apparatus.

### Tail Suspension Test

Mice were suspended for 6 min by the tail from a horizontal ring stand bar elevated to a height of 30 cm using adhesive tape. The time of immobility was recorded manually (key‐in in ANY‐maze). Mice trying to escape climbing up their tail were excluded from the study.

### Calorimetric Chamber

Mice were habituated in the testing room at 30 °C for 5 days including for 3 days in habituation cages before they were put inside the climate chamber at a constant temperature of 30 °C and humidity of 50%. Light was ON at 7am in the morning with 80 lux in the chamber, and OFF at 7pm. Animals had ad libitum access to food and water.

Oxygen and carbon dioxide sensors were calibrated with calibration gas mixtures (Calibration report: Good air equivalent: CO_2_: 0.050 ± 0.0005%, O_2_: 20.900 ± 0.1% in N_2_; CO_2_ span: CO_2_: 1.000 ± 0.001%, O_2_: 20.000 ± 0.1% in N_2_). The sample air flow was adjusted to 0.37 L min^−1^. High precision weighing stations in combination with leak‐ and spill‐proof containers recorded accurately the body weight, food, and water intake. Spontaneous activity was recorded with two levels of infrared light beam frames surrounding each cage. Recording begun from the first entrance in the chamber, but measures were considered only from the second day in the chamber. Running wheels were added in the cage from the fifth day. The habituation with running wheels lasted 2.5 days before the data were considered. Measurements were taken every 15 min and are presented here hourly or as means per hour.

### Intraperitoneal Glucose Tolerance Test (IGTT)

Mice were injected intraperitoneally with 2.0 g kg^−1^ body weight glucose after 16 h of overnight fasting. Blood glucose concentrations were measured up to 120 min after injection from the tail vein using a glucometer (Accucheck Performa, Roche, USA). All measurements were taken from 10 am‐12 noon the next day.

### Insulin Tolerance Test

Mice were injected intraperitoneally (i.p.) with 1 U kg^−1^ body weight insulin (Lilly, USA) after 4 h of fasting. Blood glucose concentrations were measured up to 120 min after injection from the tail vein using a glucometer (Accucheck Performa, Roche, USA). All measurements were taken from 10 am‐12 noon the next day.

### Liver Histopathology

Liver samples were fixed in 4% paraformaldehyde and embedded in paraffin. Histological sections were cut into 5 µm thick sections, deparaffinized, and rehydrated before staining with H&E, PSR, and MT via a Leica Autostainer XL (Leica, Germany). Frozen tissue samples for ORO staining were prepared and stained following an isopropanol‐based protocol. Images were captured using Axioscan. Z1 (Zeiss, Germany) under brightfield settings at 20× magnification. A blinded histological assessment was performed using the SAF scoring system. The steatosis score (S) ranged from 0 to 3 (S0: <5%; S1: 5–33%, mild; S2: 34–66%, moderate; S3; >67%, marked). Activity grade (A, from 0–4) is the combination of hepatocyte ballooning (0–2) and lobular inflammation (0–2). Ballooning was scored as 0 (normal polygonal hepatocytes), 1 (round, not enlarged hepatocytes, reticulated cytoplasm), and 2 (2× enlarged hepatocytes with clear cytoplasm and clumping of intermediate fibers). Lobular inflammation was scored as 0 (none), 1 (≤2 foci per 20× field), and 2 (>2 foci per^−1^ 20× field). Fibrosis scoring was as follows: stage 0 (F0): none; stage 1 (F1): 1a or 1b delicate or dense zone 3 perisinusoidal fibrosis, respectively, 1c periportal fibrosis only; stage 2 (F2): zone 3 perisinusoidal fibrosis and periportal fibrosis; stage 3 (F3): bridging fibrosis; and stage 4 (F4): cirrhosis. Portal inflammation was noted as absent or present.

### In Vivo Magnetic Resonance Imaging

Prior to in vivo imaging, animals were initially anaesthetized with 2.5–3% isoflurane in combination with medical oxygen and medical air in a dedicated mouse chamber. Isoflurane concentration was reduced following induction to 1–2% during imaging to maintain respiration between 80–90 cycles min^−1^. Animal's respiration rate and body temperature were monitored through a physiological monitoring system. Respiratory‐linked gating with a 50 ms trigger delay was used during imaging. In vivo magnetic resonance imaging (Bruker 9.4 T Biospec) was performed using a 40 mm transmit/receive body coil. High‐resolution anatomical T1‐weighted images were acquired by a Fast Low Angle Shot (FLASH) sequence with a repetition time (TR)‐337 ms, echo time 2.5 ms, flip angle (FA) 30°, averages(AV)−3, image matrix size 256 × 256 and field of view (FOV)‐ 40 × 40 mm. High‐resolution anatomical T2 weighted images were acquired using RApid imaging with Refocused Echoes (RARE) sequence with TR‐5175 ms, TE‐30 ms, FA‐90°, AV‐2, image matrix size 256 × 256 and FOV‐ 40 × 40 mm. Hepatic proton density fat fraction imaging was performed on LIDPAD (L) and control (C) mice at week 4 (*n*: *L* = 3, *C* = 3), week 8 (*n*: *L* = 5, *C* = 4) and week 16 (*n*, *L* = 4, *C* = 6) using multi‐echo Dixon (m‐Dixon) pulse sequence with parameters: TR‐12 ms, TEs‐1.85, 2.08, 2.32, 2.56, 2.80, 3.04, 3.27, 3.51 ms, FA‐5°, slices‐30, slice thickness‐1 mm, images matrix size‐ 256 × 256 and FOV‐ 40 × 40 mm. The m‐Dixon imaging data was processed and liver PDFF images were generated using the Fat‐Water Toolbox. Identical regions of interest were drawn within the liver of all animals by matching anatomical positions. Liver PDFF values were expressed as average percentages and statistical analysis (Mann–Whitney tests) was performed using SPSS software V23.

### Histological Preparation of Kidney Samples and Mesangial Expansion Index Quantification

Kidneys were fixed in buffered formaldehyde solution, processed, and embedded in paraffin. Thick tissue sections (4 µm) were stained with H&E, Periodic Acid‐Schiff (PAS) Stain Kit (ab150680), and MT stain, according to the manufacturer's protocol. All slide images were captured using a Carl Zeiss Axio Slide Scanner Z1. To measure the mesangial index, 10–15 glomeruli were identified from each kidney section. The mesangial area (PAS‐positive region) and glomerular tuft area were measured using ImageJ. The mesangial expansion index which was the ratio of mesangial area to glomerular tuft area was calculated.

### Pancreas Harvesting, H&E Staining, and Islet Size Determination

Harvested pancreatic specimens were fixed in 4% paraformaldehyde (PFA) for 16 h followed by cryoprotection in 30% sucrose for an additional 24 h. The specimens were embedded and sectioned (5 µm) in Optimal Cutting Temperature compound (OCT, Sakura, Japan), stained with H&E followed by image acquisition using a Carl Zeiss Axio Slide Scanner, Z1 at 20× magnification. Zoomed‐in high‐resolution images of the islets were documented together with a digital ruler and annotations. Islet area was calculated by demarcating islet boundaries (Image J, NIH) and size distribution were quantified with reference to the smallest and largest calculated islet area. Islet areas were then collated and evaluated on GraphPad Prism. Data were presented as the means ± SEMs (≥5 islets per animal).

### Fluorescein Isothiocyanate (FITC)‐Dextran Intestinal Permeability Assay

Mice were fasted for 4 h and given a FITC‐dextran dose (80 mg mL^−1^ in PBS) at 600 mg k^−1 ^g body weight via oral gavage. After 4 h, the mice were sacrificed, and blood was drawn via cardiac puncture. Extracted plasma was then diluted (1:5 v/v in PBS), and fluorescence was measured at excitation and emission *λ* values of 485 and 528 nm, respectively. The fluorescence intensity was then compared to the calibration curve from known concentrations of FITC‐dextran.

### Calorimetric Chamber

Mice were prehabituated in the testing room at 30 °C for 5 days, including for 3 days in habituation cages. The climate chamber was maintained at a constant temperature of 30 °C and humidity of 50%. The light was ON at 7 am with 80 lux in the chamber and OFF at 7 pm. Animals had ad libitum access to food (LIDPAD or control accordingly) and filtered water.

Oxygen and carbon dioxide sensors were calibrated with calibration gas mixtures (calibration report: good air equivalent: CO_2_: 0.050 ± 0.0005%, O_2_: 20.900 ± 0.1% in N2; CO_2_ span: CO_2_: 1.000 ± 0.001%, O_2_: 20.000 ± 0.1% in N2). The sample air flow was adjusted to 0.37 L min^−1^. High‐precision weighing stations combined with leak‐ and spill‐proof containers recorded the body weight, food intake, and water intake. Spontaneous activity was recorded with two levels of infrared light beam frames surrounding each cage. Recording began from the chamber's first entrance, but measures were considered only from the second day in the chamber. Running wheels were added to the cage from the 5th day. Habituation with running wheels lasted 2.5 days before the data were considered. Measurements were taken every 15 min and are presented here hourly or as the means per hour.

### Immunoblotting and Quantitative PCR (qPCR)

The workflow of immunoblotting and qPCR was published previously.^[^ ^40^ ^]^ For immunoblotting, anti‐squalene epoxidase and anti‐GAPDH antibodies from Santa Cruz (U.S.A.), and anti‐mouse IgG secondary antibodies from LI‐COR Biosciences (U.S.A.) were used as primary and secondary antibodies respectively. For qPCR, the nucleotide sequences of the primers are listed in Table S2 (Supporting Information).

### RNA Sequencing

Total RNA was extracted from frozen liver samples obtained from all mouse groups and patient samples with TRIzol reagent (Life Technologies, U.S.) and E.Z.N.A. HP Total RNA kit (Omega Biotek, U.S.). RNA samples were sequenced using the Illumina HiSeq platform to generate 50‐bp paired‐end reads. Raw reads were mapped to the *Mus musculus* genome assembly from Ensembl, GRCm38, or GRCh38 for humans,^[^ ^41^ ^]^ via HISAT2.^[^ ^42^ ^]^ Uniquely mapped reads were analyzed with FeatureCounts^[^ ^43^ ^]^ to produce gene count matrices, which were subjected to differential expression analysis using DESeq2.^[^ ^44^ ^]^ Genes meeting the criteria of >±1 log2‐fold‐change and adjusted *p*‐value < 0.05 were considered differentially expressed genes (DEGs). DEGs were stratified by expression and analyzed using Gene Ontology and Ingenuity Pathway Analysis (QIAGEN Inc.) to identify the top‐ranked enriched pathways. Raw sequences were deposited into GEO with accession numbers GSE159911.

### Lipid Analyses using Liquid Chromatography–Mass Spectrometry

Lipids were extracted from mouse liver tissue using liquid–liquid extraction as described^[^ ^45^ ^]^ with modifications. Briefly, 10–20 mg of tissue was mechanically homogenized in methanol: MTBE mixture using bead beating (Precellys). The lysates were further subjected to sonication in an ultrasonic bath with ice for 10 min. Phase separation was induced by adding water, followed by a 10 min incubation at room temperature. The sample was then centrifuged at 1000 × g for 10 min. The upper organic phase was collected, and the aqueous phase was re‐extracted. The pooled organic phase was dried under vacuum using a Speedvac, and the lipid extracts were stored at −80 °C prior to further analyses.

Samples were spiked with internal standards containing d7‐cholesterol (Avanti Lipids), and sterols were analyzed using atmospheric pressure chemical ionization triple quadrupole mass spectrometry (SCIEX Qtrap 6500+) with upfront liquid chromatography (Agilent Technologies). The separation of sterols was achieved using a Poroshell 120 SB‐C18 column (3.0 × 50 mm, 2.7 µm, Agilent Technologies), which was a modification of a previous method.^[^ ^46^ ^]^ For mass spectrometry analyses, the instrument was operated in multiple reaction modes. For quantitation, the area under the curve was obtained for each sterol measured and normalized to the internal standard and tissue weight. External calibration was performed for cholesterol and squalene to adjust the response factor for squalene relative to cholesterol.

### Serum Cytokine Array

Blood samples collected from the mice via cardiac were left to clot for 30 min at room temperature and then transferred to ice. The clotted samples were spun in the centrifuge at 12 000 × g for 1 min to separate the serum. The supernatant was carefully retrieved and stored. A mouse cytokine 32‐plex discovery assay was then performed by Eve Technologies (Calgary, Canada).

### Identification of Human MASLD‐Associated Gene Signature for Disease Staging

Ten MASLD liver transcriptomic datasets, which were split into training set (GSE130970, GSE162694, GSE207310, GS225740; with NAS and fibrosis stage) and validation set (GSE115193, GSE126848, GSE147304, GSE160016, GSE174478 and GSE175448; with disease staging) were obtained from GEO. The training datasets were successfully integrated by modeling the effects from batch and gender in DESeq2. For visualization, batch‐ and gender‐adjusted data was obtained using the *limma::removeBatchEffect* function.^[^ ^47^ ^]^ To identify genes associated with disease severity, a differential gene expression analysis was performed using NAS score and fibrosis stage as covariates while controlling for the batch and gender effects (*design = *≈*batch + gender + NAS score + Fibrosis stage*). Genes with a log2 fold change > ±0.1 (per unit change of NAS score) or > ±0.2 (per unit change of fibrosis stage) with an adjusted *p*‐value < 0.05 were considered MASLD‐associated DEGs. Boruta feature selection algorithm was used to shortlist gene signatures.^[^ ^22^ ^]^ Manual curation was carried out to include only genes positively correlated with disease progression and with a mouse ortholog based on biomaRT.^[^ ^48^ ^]^ This analysis resulted in a human–mouse unified MASLD transcriptomic signature comprised of 54 genes (Figure S4C, Data 2, Supporting Information).

For comparative transcriptomic between different diet‐induced MASLD models, liver transcriptomes of mice treated with MCD (GSE156918, GSE144443), CDAHFD (GSE137449), HFD (GSE222171, GSE212294, GSE198358, GSE213355, GSE180353, GSE205021), ALIOS (GSE198358), AMLN (GSE195483), and GAN (GSE225616) for varying durations were obtained from GEO. The gene counts data from these mouse datasets, the LIDPAD model, and the liver transcriptomes of in‐house MASH patient cohorts and training datasets were subjected to variance stabilizing transformation (vst). Genes with both the human and mouse orthologs were retained. ssGSEA was performed on all the samples using the unified MASLD transcriptomic signature and the enrichment scores were ranked for disease stratification and mapping for mouse MASLD severity to the human counterparts.^[^ ^49^ ^]^

### 16S rRNA Profiling

Bacterial DNA extraction was carried out on mouse feces using a QIAamp PowerFecal Pro DNA Kit (QIAGEN Inc., Hilden, Germany). The 16S rRNA V3‐V4 regions were amplified using Phusion High‐Fidelity PCR Master Mix (New England Biolabs). Libraries were generated (NEBNext UltraTM DNA Library Prep Kit) and sequenced on an Illumina MiSeq (Genewiz, Singapore). The sequenced paired‐end reads were combined using FLASH (v1.2.7), while quality control and filtering were carried out using QIIME (v1.7.0). Chimera sequences were checked and removed using the Gold database and UCHIME algorithm. Sequence analysis was performed using UPARSE (v7.0.1001) on the effective tags, and sequences with ≥97% similarity were assigned to the same operational taxonomic units (OTUs). Subsequently, the OTU sequences were compared against the SSUrRNA data of SILVA for taxonomic annotation using Mothur. Alpha and beta diversity were calculated using Qiime (v1.7.0) and exported into R for plotting using the ggplot2 and FactoMineR packages.

## Conflict of Interest

The authors declare no conflict of interest.

## Author Contributions

Z.S.L., D.C., H.S.C., and R.T. contributed equally to this work. W.W. conceived the Liver Disease Progression Aggravation Diet (LIDPAD) project. S.S.N., B.M., and W.W. developed the LIDPAD; S.S.N. conducted the proof‐of‐concept experiments. R.T. organized the animal experimentation. Z.B. and W.W. conceived and designed the metabolic and behavioral analysis. Z.S.L., D.C., H.S.C., and W.R.T. performed, and analyzed the RNA‐ and 16S‐sequencing experiments. C.B., J.Y., S.K.V., H.P., and S.S.V. conducted MRI imaging and PDFF analysis. Z.S.L., D.C., C.H.S., R.T., W.R.T., N.B.E.S., Z.Y., J.C., A.S.N., Y.S.Y., M.I.G.V., N.A.T., D.L.L., D.X.E.L., and M.C. performed the animal experiments, histology, and biochemical assays. A.W., N.C.L., and M.D.M. scored the liver biopsies as clinical pathologists. M.S.K. prepared and analyzed the histology of the kidney. V.S.Y.T. and Y.A. prepared and analyzed the histology of the islet cells. X.L.G. processed and analyzed the lipids. J.Q., Y.L., H.H., C.C., and L.L. performed histological staining, scoring, and RNA sequencing on the patient samples. Z.S.L., D.C., C.H.S., N.S., and W.W. wrote the manuscript with inputs from all authors.

## Supporting information

Supporting Information

Supplemental Data 1

Supplemental Data 2

Supplemental Data 3

## Data Availability

High‐throughput sequencing data can be found in the GEO database (GSE159911). All other data needed to evaluate the conclusions in the paper are present in the paper and/or the Supporting Information. The R scripts used for bioinformatic analysis are available at Github (https://github.com/chenghongsheng/Angptl4‐in‐NAFLD‐immunopathologies).
