# Supplementary material for: The LIDPAD Mouse Model Captures the Multisystem Interactions and Extrahepatic Complications in MASLD
Source: Adv Sci (Weinh). 2024 Jul 1;11(35):2404326. doi: 10.1002/advs.202404326 (PMC11425234; doi:10.1002/advs.202404326)
Supplement: Supplementary file 1 — Supporting Information [file ADVS-11-2404326-s002.pdf]

Supporting Information

The LIDPAD mouse model captures the multisystem interactions and extrahepatic complications in MASLD.

Zun Siong LOW[1†], Damien CHUA[1†], Hong Sheng CHENG[1†], Rachel TEE[1†], Wei Ren TAN[1], Christopher BALL[2], Norliza Binte Esmail SAHIB[1], Ser Sue NG[1], Jing QU[3], Yingzi LIU[4], Haiyu HONG[5], Chaonong CAI[5], Nandini C. L. RAO[6], Aileen WEE[7], Mark D. MUTHIAH[8,9,10], Zoë BICHLER[1‡], Barbara MICKELSON[11], Mei Suen KONG[1], Vanessa S.Y. TAY[1], Zhuang YAN[1], Jiapeng CHEN[1], Aik Seng NG[12], Yun Sheng YIP[1], Marcus Ivan Gerard VOS[1], Dao Liang LIM[13], Nicole Ashley TAN[13], Debbie Xiu En LIM[1], Manesh CHITTEZHATH[1], Jadegoud YALIGAR[2,14], Sanjay Kumar VERMA[2], Harish POPTANI[15], Xue Li GUAN[1], S.Sendhil VELAN[2,9,14], Yusuf ALI[1,16], Liang LI[17], Nguan Soon TAN[1,13] and Walter WAHLI[1,18,19*]*

[1.] Lee Kong Chian School of Medicine, Nanyang Technological University Singapore, Clinical Sciences Building, 11 Mandalay Road, 308232 Singapore, Singapore

[2.] Metabolic Imaging Group, Institute of Bioengineering and Bioimaging, Agency for Science Technology and Research (A*STAR), 11 Biopolis Way, Singapore 138667

[3.] Department of Pathogen Biology, Shenzhen Center for Disease Control and Prevention, Shenzhen, China.

[4.] Peking University Shenzhen Hospital, Shenzhen, China.

[5.] The Fifth Affiliated Hospital of Sun Yat-sen University, 52 Mei Hua East Road, Zhuhai, 519000 China

[6.] Department of Pathology, Tan Tock Seng Hospital, 11 Jalan Tan Tock Seng, 308433 Singapore, Singapore

[7.] Department of Pathology, National University Hospital, 5 Lower Kent Ridge Rd, 119074 Singapore, Singapore.

[8.] Department of Medicine, Yong Loo Lin School of Medicine, National University of Singapore, Singapore 117597, Singapore

[9.] Division of Gastroenterology and Hepatology, Department of Medicine, National University Hospital, Singapore 119074, Singapore.

[10.] National University Centre for Organ Transplantation, National University Health System, Singapore 119074, Singapore.

[11] ENVIGO, Madison, WI 53713, USA
[12] Radcliffe Department of Medicine, John Radcliffe Hospital, University of Oxford, Oxford, United Kingdom
[13] School of Biological Sciences, Nanyang Technological University Singapore, 60 Nanyang Drive, 637551 Singapore, Singapore.
[14] Singapore Institute for Clinical Sciences, A*STAR, 30 Medical Drive, Singapore 117609.
[15] Centre for Preclinical Imaging, Institute of Systems, Molecular & Integrative Biology, University of Liverpool, Biosciences Building, Crown Street, Liverpool, L69 7BE
[16] Singapore Eye Research Institute (SERI), Singapore General Hospital, Singapore.
[17] Department of Pharmacology, School of Medicine, Southern University of Science and Technology, Shenzhen, China.
[18] Institut national de recherche pour l'agriculture, l'alimentation et l'environnement (INRAE), Toxalim (Research Centre in Food Toxicology), 180 Chemin de Tournefeuille, 1331 Toulouse, France.
[19] Center for Integrative Genomics, Université de Lausanne, Le Génopode, 1015 Lausanne, Switzerland.

†These authors contributed equally.
*Corresponding authors: N.S.Tan (nstan@ntu.edu.sg); W.Wahli (walter.wahli@ntu.edu.sg)

Supplemental Table and Data

Table S1. Nutritional information of LIDPAD, high-fat diet and control diet

Table S2. List of qPCR primers

Supplementary Data 1. Differentially expressed genes (DEGs) associated with NAFLD activity score and fibrosis stages from the meta-analysis of four human liver transcriptome datasets.

Supplementary Data 2. Unified MASLD transcriptomic signature from human and mouse disease staging.

Supplementary Data 3. Union of all DEGs obtained from MASH vs Control, MASH_R vs Control and MASH_R vs MASH.

Supplemental Tables

Table S1. Nutritional information of LIDPAD, high-fat diet and control diet

LIDPAD (Liver Disease Progression Aggravation Diet, Teklad Custom Diet, 1% cholesterol, Envigo)	
Macronutrient Information	% kcal
**Protein**	11.5
**Carbohydrate**	45.2
**Fat**	43.3

Kcal/g: 4.6

High-fat diet (D12451, SYSE Bio)	
MacroNutrient Information	% kcal
**Protein**	20
**Carbohydrate**	35
**Fat**	45

Kcal/g: 4.73

Control (Teklad Custom Diet, Envigo)	
MacroNutrient Information	% kcal
**Protein**	13.7
**Carbohydrate**	75.9
**Fat**	10.3

Kcal/g: 3.6

Table S2. List of qPCR primers

Primers	Accession number	Forward primer (5’ to 3’)	Reverse primer (3’ to 5’)
18s	NR_003278	GTA ACC CGT TGA ACC CCA TT	CCA TCC AAT CGG TAG TAG GG
Il6	NM_031168	TAG TCC TTC CTA CCC CAA TTT C	TTG GTC CTT AGC CAC TCC TTC
Cxcl10	NM_021274	CCA AGT GCT GCC GTC ATT TTC	GGC TCG CAG GGA TGA TTT CAA
Ccl20	NM_016960	GCC TCT CGT ACA TAC AGA CGC	CCA GTT CTG CTT TGG ATC AGC
Col1a1	NM_007742	CTG GCG GTT CAG GTC CAA T	TTC CAG GCA ATC CAC GAG C
Col3a1	NM_009930	ATA CCC GGA ACA CGA GGT C	CAT CTT CGC CCT TAG GTC CTG
Col4a3	NM_007734	CAG GGG ATT CAA TGG AAA AGG T	TAT CAC GCT ATC GCC CAT GTG

A

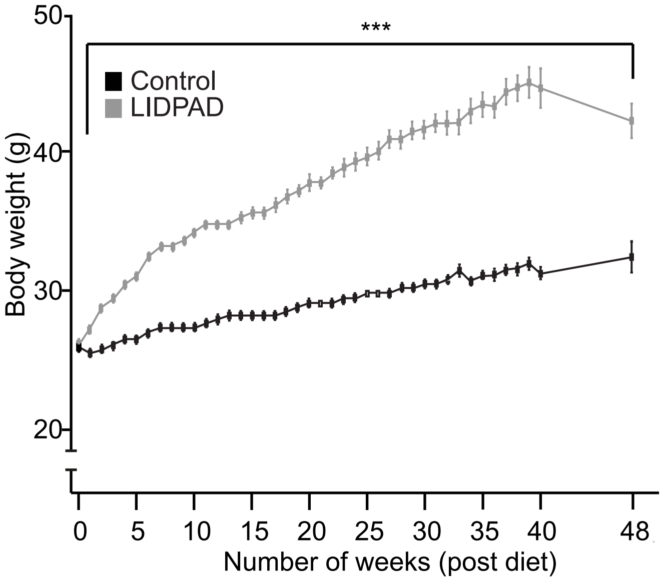

Duration on diet	Number of animals	
	Control	LIDPAD
1	84	90
4	64	71
8	51	57
12	38	47
16	33	37
32	30	34
36	20	25
40	13	17
48	8	9

B

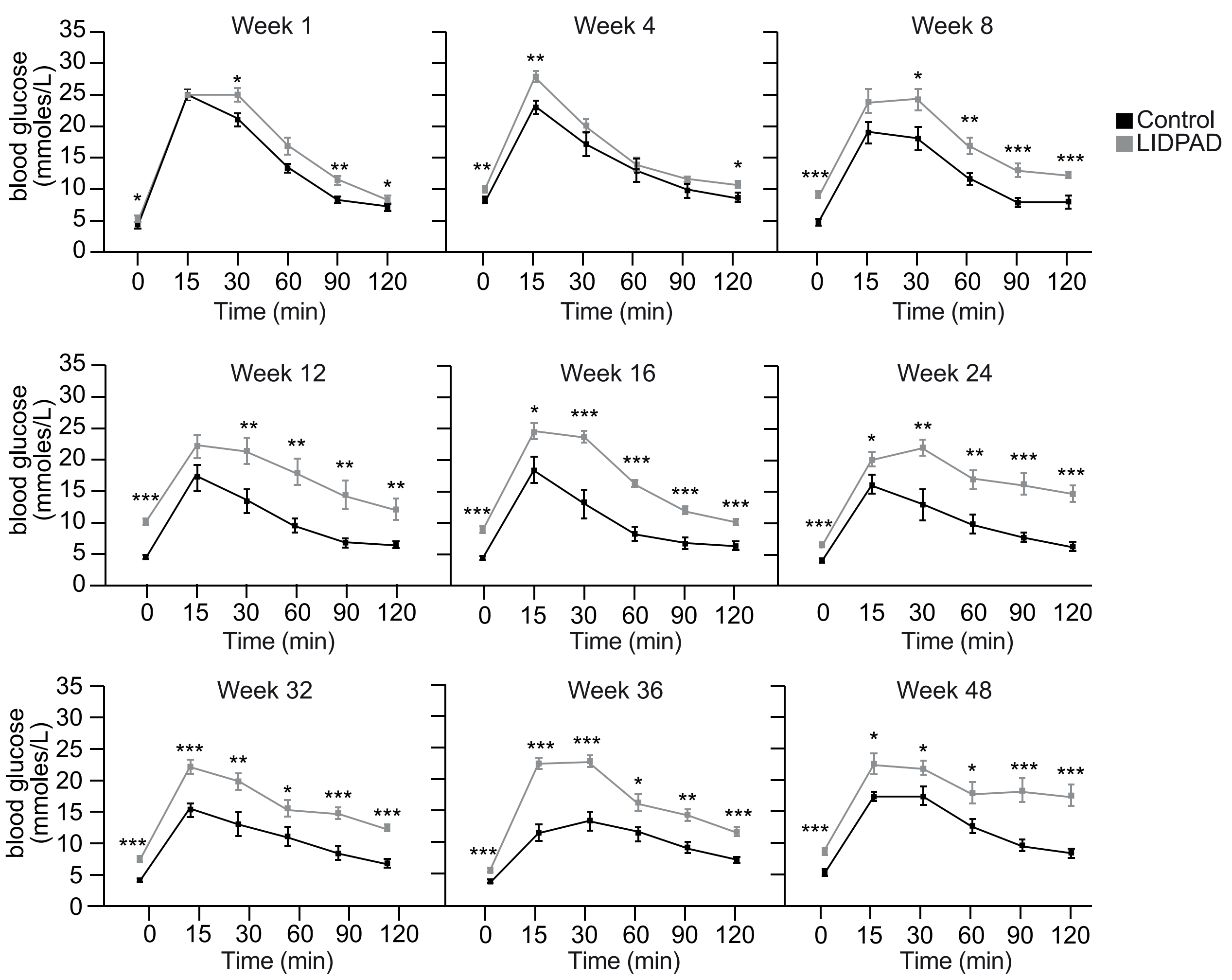

C

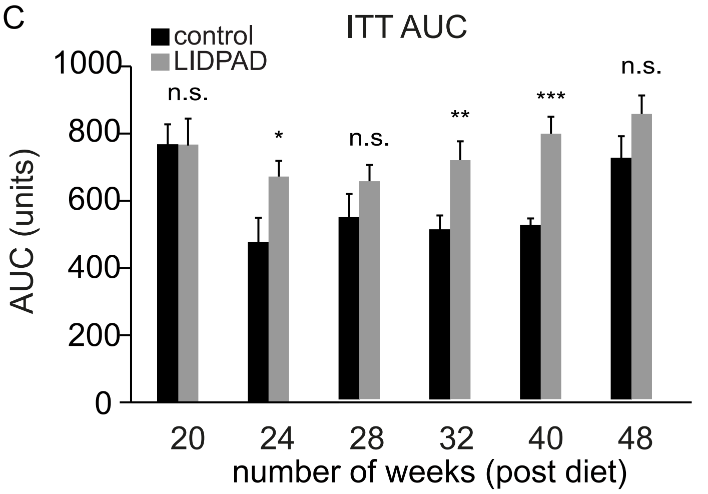

Supplemental Figure Legends

Figure S1: Metabolic parameters of control and LIDPAD mice.

A. Change in weight (left) of mice fed the control diet and LIDPAD and the number of animals (right) measured at each time point for 48 weeks**.**

B. Intraperitoneal glucose tolerance test (IGTT) curves for the indicated timepoints.

C. Area under curve (AUC) of insulin tolerance test (ITT) of LIDPAD- and control-fed mice from weeks 20 to 48.

n = 7-10 per group. Data are expressed as the means ± SEMs. ***$p<0.001$, **$p<0.01$, *$p<0.05$ (unpaired t test, ANOVA Welch's t test or ANCOVA test when appropriate, followed by post hoc comparisons).

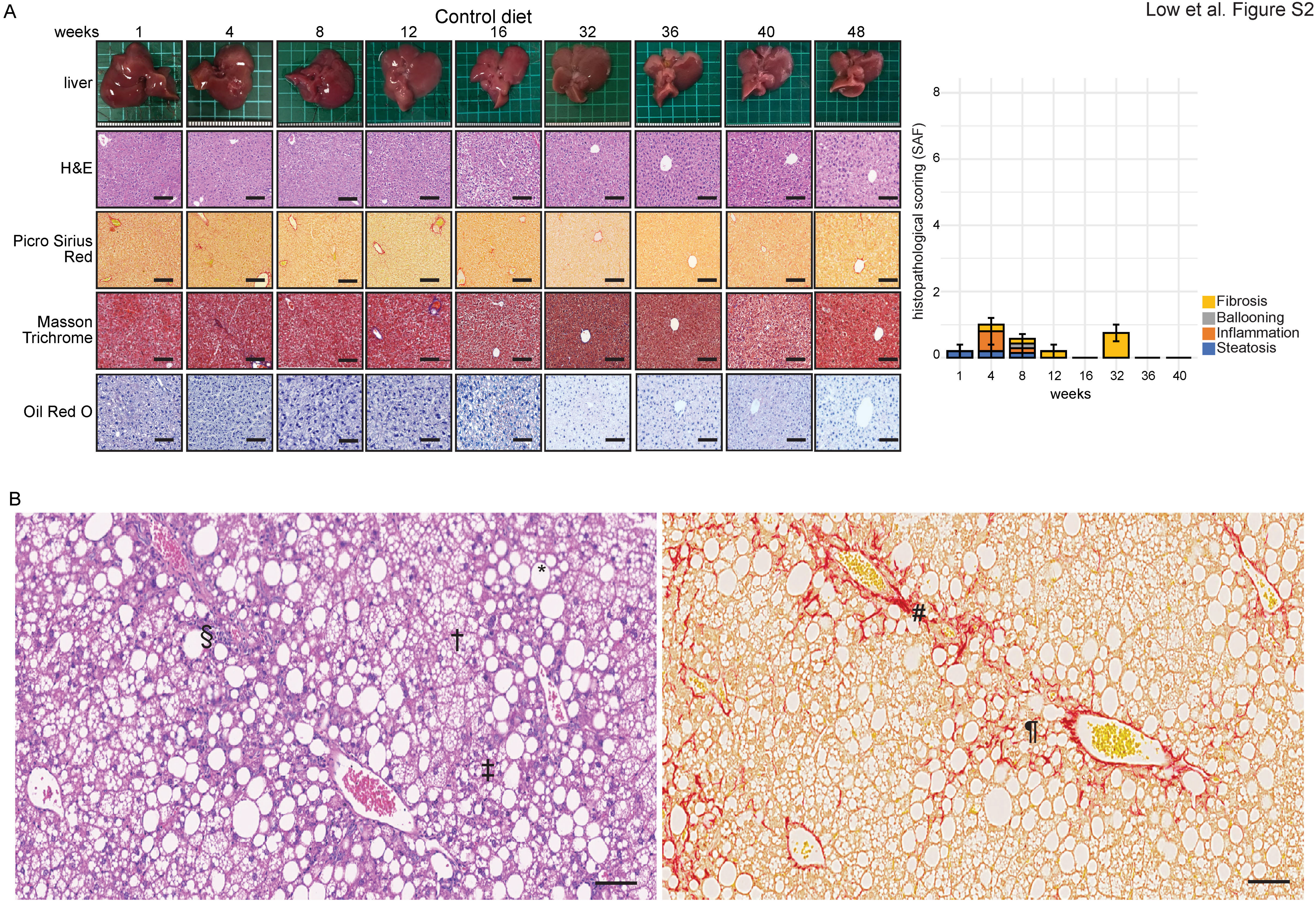
A
Control diet
weeks
1
4
8
12
16
32
36
40
48
liver
H&E
Picro Sirius Red
Masson Trichrome
Oil Red O
histopathological scoring (SAF)
8
6
4
2
0
1
4
8
12
16
32
36
40
weeks
Fibrosis
Ballooning
Inflammation
Steatosis
B
§
†
*
‡
#
¶

Figure S2: Representative macroscopic and microscopic images of the livers obtained from control and LIDPAD mice.

A. Histological sections were stained with hematoxylin and eosin (H&E) to show general liver features, picrosirius red, and Masson's Trichrome to highlight collagen deposition, and oil red O to detect the presence of lipids. Scale bar represents 100 μm. A bar plot shows histological SAF scores of the livers from control mice at the indicated weeks post-feeding (right). Data are expressed as the means ± SEM.

B. Representative histological images of mouse livers fed LIDPAD for 16 weeks, stained with H&E and PSR to indicate the hepatic pathological features. * indicates macrovesicular steatosis; † indicates microvesicular steatosis; ‡ indicates ballooned hepatocytes; § indicates the region of lobular inflammation; ¶ indicates the region of pericellular fibrosis; # indicates periportal fibrosis. The scale bar represents 100 μm.

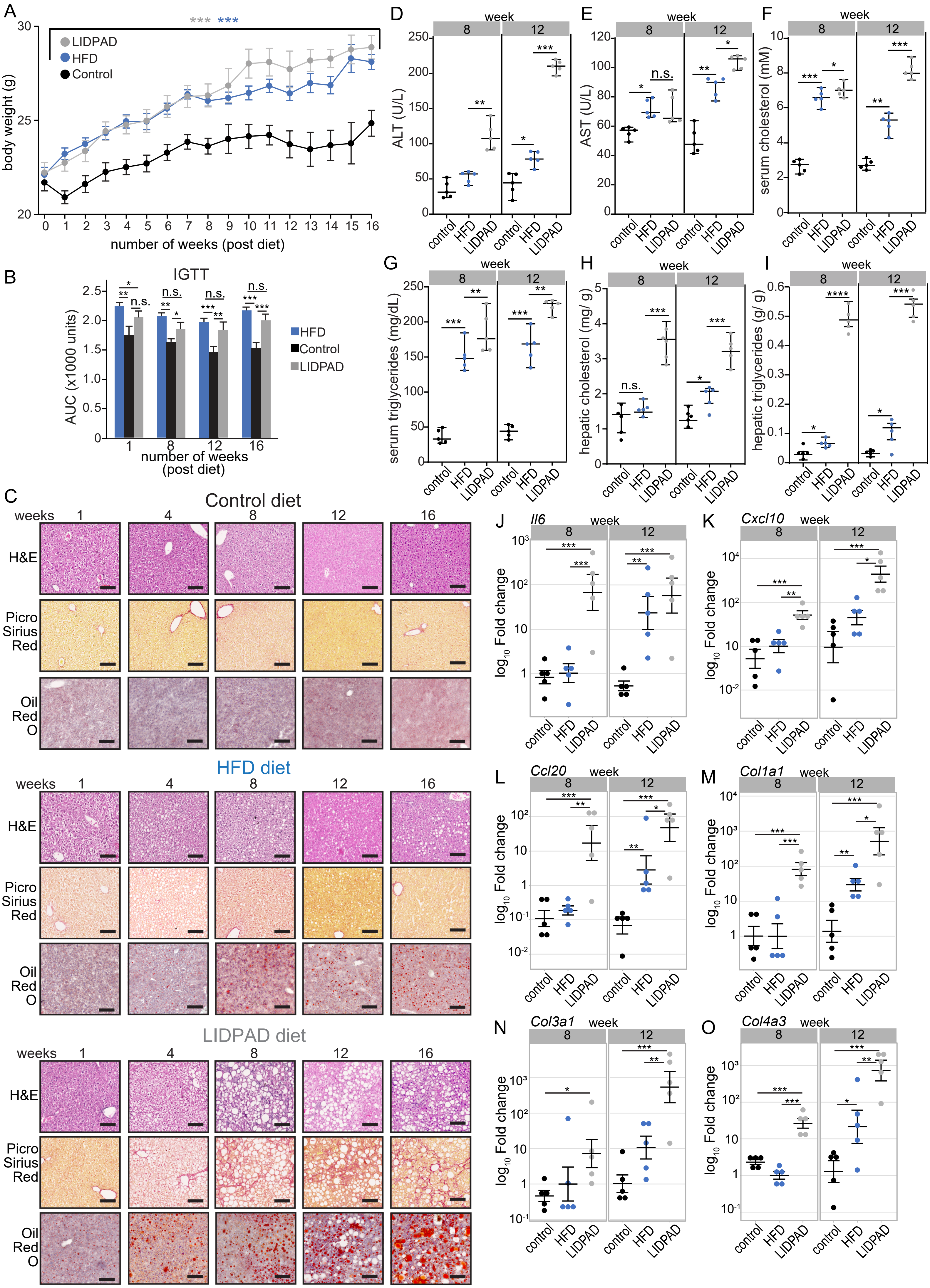
A
LIDPAD
HFD
Control
body weight (g)
number of weeks (post diet)
B
IGTT
AUC (x1000 units)
HFD
Control
LIDPAD
number of weeks
(post diet)
C
Control diet
HFD diet
LIDPAD diet
weeks
H&E
Picro Sirius Red
Oil Red O
D
ALT (U/L)
E
AST (U/L)
F
serum cholesterol (mM)
G
serum triglycerides (mg/dL)
H
hepatic cholesterol (mg/ g)
I
hepatic triglycerides (g/ g)
week
control
HFD
LIDPAD
J
Il6
K
Cxcl10
L
Ccl20
M
Col1a1
N
Col3a1
O
Col4a3
log10 Fold change

Figure S3. Metabolic parameters of mice fed on high-fat diet (HFD).

A. Change in weight of mice fed with either Control, LIDPAD or High-Fat diet (HFD) measured at each time point for 16 weeks at thermoneutral housing.

B. Area under the curve (AUC) of intraperitoneal glucose tolerance test (IGTT) curves for the indicated timepoints.

C. Representative microscopic images of the livers obtained from HFD mice. Histological sections were stained with hematoxylin and eosin (H&E), picrosirius red and Oil red O. Scale bar represents 100 µm. A bar plot (right) shows histological SAF scores of the livers from HFD mice at the indicated weeks post-feeding.

D-I. Liver function test consisting of alanine aminotransferase (ALT) **(D)** and aspartate aminotransferase (AST) **(E),** and total cholesterol and triglyceride levels in the serum **(F-G)** and livers **(H-I)** of control-, HFD- and LIDPAD-fed mice at 8 and 12 weeks.

J-O. Quantitative PCR (qPCR) of inflammatory (*Il6, Cxcl10* and *Ccl20*) and fibrosis (*Col1a1, Col3a1* and *Col4a3*) markers of control-, HFD- and LIDPAD-fed mice at 8 and 12 weeks. *18s* was used as the endogenous control gene for normalization.

For **A** and **B**, n = 12 per group; for **D-O**, n=5 per group per timepoint. Data are expressed as the means ± SEMs. ****p<0.0001, ***p<0.001, **p<0.01, *p<0.05 (unpaired t test, ANOVA Welch's t test or ANCOVA test when appropriate, followed by post hoc comparisons).

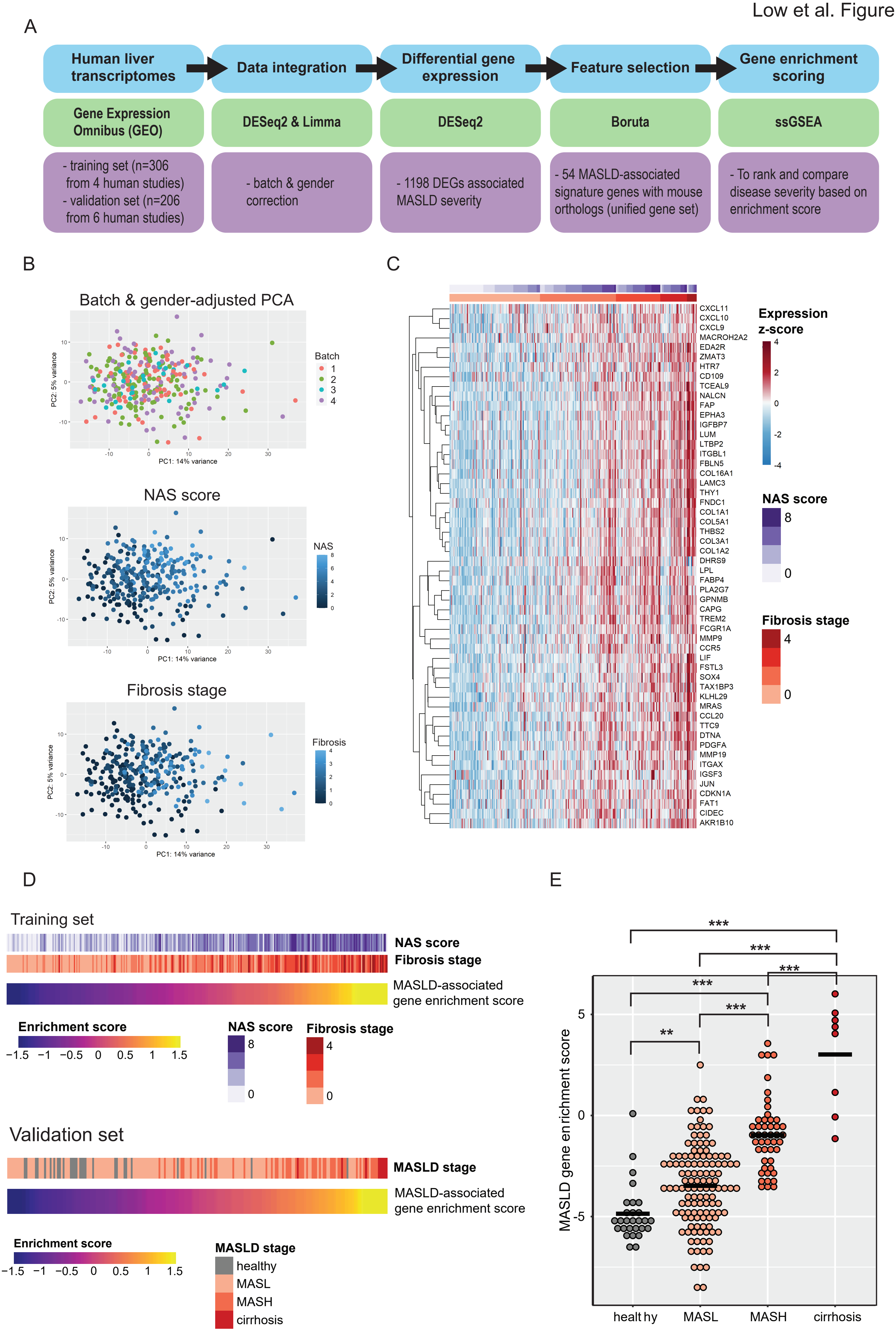
A
Human liver transcriptomes
Data integration
Differential gene expression
Feature selection
Gene enrichment scoring
Gene Expression Omnibus (GEO)
DESeq2 & Limma
DESeq2
Boruta
ssGSEA
- training set (n=306 from 4 human studies)
- validation set (n=206 from 6 human studies)
- batch & gender correction
- 1198 DEGs associated MASLD severity
- 54 MASLD-associated signature genes with mouse orthologs (unified gene set)
- To rank and compare disease severity based on enrichment score
B
Batch & gender-adjusted PCA
PC2: 5% variance
PC1: 14% variance
Batch
1
2
3
4
NAS score
NAS
Fibrosis stage
Fibrosis
C
CXCL11
CXCL10
CXCL9
MACROH2A2
EDA2R
ZMAT3
HTR7
CD109
TCEAL9
NALCN
FAP
EPHA3
IGFBP7
LUM
LTBP2
ITGBL1
FBLN5
COL16A1
LAMC3
THY1
FNDC1
COL1A1
COL5A1
THBS2
COL3A1
COL1A2
DHRS9
LPL
FABP4
PLA2G7
GPNMB
CAPG
TREM2
FCGR1A
MMP9
CCR5
LIF
FSTL3
SOX4
TAX1BP3
KLHL29
MRAS
CCL20
TTC9
DTNA
PDGFA
MMP19
ITGAX
IGSF3
JUN
CDKN1A
FAT1
CIDEC
AKR1B10
Expression z-score
NAS score
Fibrosis stage
D
Training set
NAS score
Fibrosis stage
MASLD-associated gene enrichment score
Enrichment score
−1.5 −1 −0.5 0 0.5 1 1.5
Validation set
MASLD stage
healthy
MASL
MASH
cirrhosis
E
MASLD gene enrichment score
healthy
MASL
MASH
cirrhosis
***
**

Figure S4: Meta-analysis of human MASLD livers to establish MASLD-associated transcriptomic signature.

A. Workflow and analytical pipelines using to establish a human transcriptomic-guided staging approach based on liver RNAseq meta-analysis.

B. Principal component analysis of the integrated MASLD liver transcriptomes. Each data point is color-coded based on the batch (top), NAS score (middle) and fibrosis stage (bottom) to demonstrate the successful data integration and spread of the liver transcriptomes according to disease severity.

C. Heatmap of human-mouse unified MASLD transcriptomic signature (54 genes) in MASLD patient cohorts in **B**.

D. Patient stratification of the training set (patient cohorts in **B**; top) and validation set (independent patient cohorts from six other studies; bottom) based on ranked enrichment scores of the unified MASLD transcriptomic signature. The NAS score and fibrosis stage or disease staging of each individual was retrieved from the respective study and annotated.

E. Dot plot of the enrichment scores at different MASLD stages from the validation set. Black bars indicate the mean of scores of each group. ***$p<0.001$, **$p<0.01$ (one-way ANOVA followed by post hoc pairwise comparisons with FDR adjustment).
